# SPA: a peptide antagonist that acts as a cell-penetrating peptide for drug delivery

**DOI:** 10.1080/10717544.2019.1706669

**Published:** 2019-12-24

**Authors:** Jingjing Song, Sujie Huang, Zhengzheng Zhang, Bo Jia, Huan Xie, Ming Kai, Wei Zhang

**Affiliations:** a Institute of Pharmacology, Key Laboratory of Preclinical Study for New Drugs of Gansu Province, School of Basic Medical Sciences, Lanzhou University, Lanzhou, China;; b Institute of Biochemistry and Molecular Biology, School of Life Sciences, Lanzhou University, Lanzhou, China;; c Institute of Physiology, Key Laboratory of Preclinical Study for New Drugs of Gansu Province, School of Basic Medical Sciences, Lanzhou University, Lanzhou, China

**Keywords:** Peptide antagonist, cell penetrating peptide, camptothecin delivery, gene delivery

## Abstract

Although cell-penetrating peptides (CPPs) has been proven to be efficient transporter for drug delivery, ideal peptide vectors for tumor therapy are still being urgently sought. Peptide antagonists have attracted substantial attention as targeting molecules because of their high tumor accumulation and antitumor activity compared with agonists. SPA, a derivative of substance P, is a potent antagonist that exhibits antitumor activity. Based on the amino acid composition of SPA, we speculate that it can translocate across cell membranes as CPPs do. In this study, our results demonstrated that SPA could enter cells similarly to a CPP. As a vector, SPA could efficiently deliver camptothecin and plasmids into cells. In addition, our results showed that SPA exhibited low toxicity to normal cells and high enzymatic stability. Taken together, our results validated the ability of SPA for efficient drug delivery. More importantly, our study opens a new avenue for designing ideal CPPs based on peptide antagonists.

## Introduction

Tumor chemotherapy still remains a great challenge despite remarkable progress in the development of antitumor drugs in recent years. For example, severe adverse effects, inefficient tumor accumulation and the emergence of multidrug resistance have resulted in a low therapy index for many hydrophobic small-molecule antitumor drugs. The rapid development of molecular and cellular biology has fostered the production of several new classes of highly potent biomolecular drugs, including nucleic acids, proteins and peptides. In contrast to small-molecule drugs that can translocate across the cell membrane via direct diffusion, biomolecular drugs have low permeability across the lipid bilayer due to their hydrophilic nature and relatively large molecular weight. Therefore, it would be desirable to develop highly efficient vectors that possess the capacity to deliver small-molecule drugs or biomolecular drugs into tumor tissues and cells. Among the developed vectors, cell-penetrating peptides (CPPs) are promising candidates due to their intrinsically efficient internalization, typically low toxicities, and easy synthesis and modification (Svensen et al., 2012; Dissanayake et al., 2017; Komin et al., 2017; Araste et al., 2018). During recent decades, extensive research has clearly validated the capacity of CPPs to serve as efficient transporters for the local and systemic delivery of various therapeutic agents, including small drugs, peptides, proteins and nucleic acids (Koren & Torchilin, 2012; Guidotti et al., 2017; Araste et al., 2018). Nonetheless, ideal peptide vectors for tumor therapy are still being urgently sought.

CPPs are short peptides that can efficiently translocate into cells (Milletti, 2012; Kauffman et al., 2015). Generally, CPPs are categorized into three major classes: cationic CPPs, amphipathic CPPs, and hydrophobic CPPs (Milletti, 2012; Wang et al., 2014). Although CPPs have gained much attention as potent transporters, their major drawbacks for *in vivo* application are short half-life *in vivo*, lack of cell and tissue specificity and possible toxic side effects (Jarver et al., 2010; Wang et al., 2014; Guidotti et al., 2017). Many effective strategies have been adopted to improve the applications of CPPs, such as the introduction of unnatural amino acids to improve their enzyme stability and designing activatable CPPs to increase their specificity (Jiang et al., 2004; Mickan et al., 2014; Wang et al., 2014).

Peptide agonists have been extensively used as targeting molecules to selectively deliver drugs and imaging agents into tumor cells because they can be internalized via receptor-mediated endocytosis (Majumdar & Siahaan, 2012; Sun et al., 2017). In contrast to agonists, antagonists are not internalized by tumor cells even though they also bind to receptors, and consequently antagonists were originally believed to be unsuitable for tumor-targeted drug delivery (Sun et al., 2017). However, an increasing number of studies have demonstrated that antagonists are more suitable as tumor targeted molecules than agonists, probably due to their ability to bind more receptor sites, slower dissociation rate from the receptor and stronger resistance to membrane-bound enzymes (Ginj et al., 2006; Cescato et al., 2008; Sun et al., 2017). Substance P (SP, RPKPQQFFGLM-NH_2_), a neurokinin-1 (NK1) receptor agonist, is one of the most extensively studied neuropeptides (Datar et al., 2004; Steinhoff et al., 2014). In our previous study, we confirmed that SP can selectively deliver conjugated camptothecin into tumor cells with high expression of the NK1 receptor (Song et al., 2011b). Simultaneously, we found that an SP-camptothecin conjugate could enter CHO cells with non-NK1 receptor expression, indicating that SP can enter cells via receptor-independent endocytosis as CPPs do. However, the uptake efficiency of SP as a CPP leaves much to be desired. SPA ([D-Arg^1^, D-Trp^5,7,9^, Leu^11^]Substance P), a derivative of SP, is an antagonist that inhibits signal transduction and cell proliferation more potently than [Arg^6^, D-Trp^7,9^, MePhe^8^]Substance P, which has entered into a phase I clinical trial (Seckl et al., 1997; Guha et al., 2005). Based on the amino acid composition of SPA compared with that of SP, we speculate that SPA should have greater cell-penetrating activity. Therefore, in the present study, we evaluated the translocation efficiency of SPA as well as its application potential in the delivery of small molecular drugs and nucleic acids.

## Materials and methods

### Synthesis of peptides and conjugate

All peptides were synthesized using standard Fmoc solid phase peptide synthesis strategy. The fluorescein moiety (FITC) was attached to the N-terminus via an aminohexanoic acid spacer by treating a resin-bound peptide (0.1 mmol) with FITC (0.1 mmol), and diisopropyl ethyl amine (0.5 mmol) in DMF for 12 h. Stearic acid was coupled as a Fmoc amino acid. SPA-camptothecin conjugate was synthesized as described previously (Henne et al., 2006). All crude peptides were purified by reversed phase high performance liquid chromatography (RP-HPLC) on a C18 column. Purity analysis was checked by analytical RP-HPLC (Waters), and the peptides were eluted using a liner gradient of 5–95% acetonitrile in 0.1% trifluoroacetic acid at a flow rate of 1 mL/min within 30 min on a C18 column (Waters XBridge Columns, 10 μM, 4.6*250 mm). The retention time of each peptide was determined when the peak was at its maximum height. Identities of the synthetic peptides were confirmed by ESI-MS. The sequences of all peptides and conjugates were shown in the Supplymentry Table S1. All peptides and conjugates were dissolved in dimethyl sulfoxide (DMSO) with a final concentration of 10 mM.

### Confocal fluorescence microscopy

CHO cells were cultured in a glass-bottomed culture dish 24 h before treatment. After washing with PBS, the cells were incubated with 10 μM FITC labeled peptides or SPA-CPT conjugate for 1 h. To observe the uptake of stearyl-SPA/plasmid complexes, 0.5 μg of Cy5-labeled pGL3 plasmids were mixed with peptides at N/P ratio of 2 in 50 μL water. After incubating 30 min at 37 °C, the complexes were diluted to a 500 μL final volume using serum free DMEM, and treats CHO cells for 4 h. Cy5-labeled pGL3 plasmid was obtained following a procedure as described by the manufacturer (Label IT Tracker Kit, Mirus). Thereafter, cells were stained with 10 μg/mL DAPI for 10 min and images were taken with confocal microscope (Zeiss LSM710).

### Flow cytometry assays

CHO cells were treated with 10 μM FITC labeled peptides for 1 h, and then the cells were harvested and finally resuspended in PBS after washing twice with PBS. The fluorescence of cells was analyzed on a FACS Caliber. To explore the uptake mechanism of SPA, CHO cells were preincubated at 4 °C and then treated with 10 μM FITC-labeled SPA at 4 °C or 37 °C for 1 h. In experiments with chloroquine, CHO cells were incubated with FITC-labeled SPA and 100 μM chloroquine at 37 °C for 1 h. In experiments with endocytosis inhibitor, cells were pretreated with 10 μg/mL chlorpromazine, 50 μM amiloride or 5 mM methyl-β-cyclodextrin at 37 °C for 0.5 h and then treated with 10 μM FITC-labeled SPA for 1 h. Cells were harvested and analyzed by a FACS Caliber (BD).

### Molecular dynamics simulations

All simulations reported in this work were performed according to our previous method (Song et al., 2013). SPA with final structure after 100 ns simulation in water was solvated in the water phase close to the monolayers of an equilibrated bilayer. This system was simulated for 200 ns.

### Enzymatic stability of peptides

The enzymatic stability of SPA toward trypsin and mice serum was determined following a procedure reported in our previous study (Song et al., 2013). A portion of 15 μL peptide stock solution (10 mM) was added to 285 μL trypsin solution (10 μg/mL) or 100% serum. Incubations were carried out at 37 °C for 0, 0.5, 1, 2 and 3 h in triplicate. A portion of 40 μL aliquots were diluted with 80 μL water/acetonitrile (60:40 v/v) containing 1% TFA and analyzed by RP-HPLC (Waters). The peptides were eluted using a liner gradient of 5–95% acetonitrile in 0.1% trifluoroacetic acid at a flow rate of 1 mL/min within 30 min on a C18 column (Waters XBridge Columns, 10 μM, 4.6*250 mm). The remaining full-length peptide concentration was normalized with respect to the initial concentration.

### Lactate dehydrogenase (LDH) leakage assays

The LDH leakage was measured using the Cytotoxicity Detection Kit (Roche). CHO cells were seeded at 1 × 104 cells/well in 96-well plate 24 h before treatment. After treatment with SPA at various concentrations for 1 h, 40 μL medium was transferred to a 96-well plate and incubated for 15 min with 40 μL reaction mixture, followed by 20 μL stop solution. Fluorescence was measured using microplate reader (Thermo scientific) at 490 nm. Untreated cells were defined as no leakage and 100% leakage was defined as total LDH release by lysing cells in 0.2% Triton X-100.

### Hemolysis assays

Erythrocytes were obtained from freshly collected mice blood and resuspended in PBS to 4% (V/V). After 100 μL the erythrocyte suspension being seeded in 96-well plate, the cells were incubated with 100 μL peptides solution at different concentrations for 1 h at 37 °C. The plates were centrifuged at 1000 g for 10 min and the released hemoglobin was determined using microplate reader (Thermo scientific) at 450 nm. PBS and 0.1% Triton X-100 were used as agents for 0 and 100% hemolysis, respectively.

### MTT assays

CHO cells were seeded at 5 × 10^3^ cells/well in 96-well plate 24 h before treatment. Cells were treated with SPA at various concentrations for 24 h. The cytotoxicity of SPA was determined by MTT assay. The absorbance was determined using microplate reader (Thermo scientific) at 570 nm.

The antiproliferative effects of SPA-CPT conjugate was also determined by MTT assay. CHO, MDA-MB-231and U251 cells were seeded at 5 × 10^3^ cells/well in 96-well plates 24 h before treatment, respectively. After being washed, cells were treated with CPT, SPA and SPA-CPT for 1 h. After washing with medium to remove the remaining agents, the cells were cultured for 72 h and the antiproliferative effects of the relevant agents were determined by the MTT assay.

### Gel retardation assay

DNA condensation was evaluated using a gel retardation assay. Briefly, the peptide/pGL3 plasmid complexes were prepared by adding appropriate volumes of peptide solution to 0.5 μL pGL3 plasmid (200 ng/μL in water). After incubation for 30 min at 37 °C, the complexes were loaded on the 0.8% (W/V) agarose gel and imaged by staining the gel with EtBr.

### Transmission electron microscopy (TEM)

0.5 μg of plasmids were mixed with peptides at N/P ratio of 2 in 50 μL water. After incubating 30 min at 37 °C, the complexes were dropped onto a copper grid, and subsequently stained by 0.2% (W/V) phosphotungstic acid solution. The morphologies of complexes were observed by TEM (JEM-1230).

### *In vitro* plasmid transfection assays

CHO cells were seeded at 1 × 10^5^ cells/well in 24-well plate 24 h before treatment. 0.5 μg of pGL3 plasmids were mixed with peptides at different charge ratios in 50 μL water. After incubating 30 min at 37 °C, the complexes were diluted to a 500 μL final volume using serum free DMEM. Cells were treated with peptide/pGL3 plasmid complexes for 4 h, and then the medium was replaced with 1 mL DMEM containing 10% FBS. After 24 h of incubation, luciferase activity was measured as described previously (Zhang et al., 2013b).

### Statistical analysis

Experiments were performed three times and the data were expressed as means ± SEM. Statistical analysis was performed using Student’s *t* tests. *p* < .05 was considered to be indicative of statistical significance.

## Results and discussion

### Cellular uptake efficiency of SPA

Although a large number of cell-penetrating peptides have been identified in recent decades, continued efforts have been devoted to obtaining ideal CPPs with high cellular uptake efficiency, cell specificity and low toxicity for clinical application. To this end, several strategies have been employed successfully, such as conformational constraints, the systematic alteration of physicochemical properties and the development of activatable CPPs (Copolovici et al., 2014; Wang et al., 2014; Peraro & Kritzer, 2018). With broad and deep studies on the structure-activity relationships of CPPs, hydrophobicity has been deemed as an important factor for enhancing the uptake efficiency of CPPs (Gupta et al., 2011; Jones & Sayers, 2012; Kabelka & Vacha, 2018). In our previous work, hydrophobic antitumor drug CPT could significantly increase the membrane-lytic activity and cell-penetrating activity of TAT after the attachment to the N-terminus of TAT (Song et al., 2015). Among the hydrophobic amino acids, tryptophan shows the strongest preference for interaction with the phospholipid bilayer and performs important anchoring functions in most membrane proteins (Yau et al., 1998). Tryptophan has been reported to contribute to enhance the cellular efficiency of CPPs by increasing hydrophobic interaction of peptides with membrane bilayers (Rydberg et al., 2012; Jobin et al., 2015). Compared with SP, SPA should exhibit high hydrophobicity due to the introduction of three D-tryptophan residues and one leucine residue. The retention time on RP-HPLC can more accurately reflect the actual hydrophobicity of peptides (Kim et al., 2005). Our results showed that the retention time of SP was 16.21 min and that of SPA was 20.98 min, confirming that SPA was more hydrophobic than SP, indicating a stronger membrane interaction.

**Figure 1.**
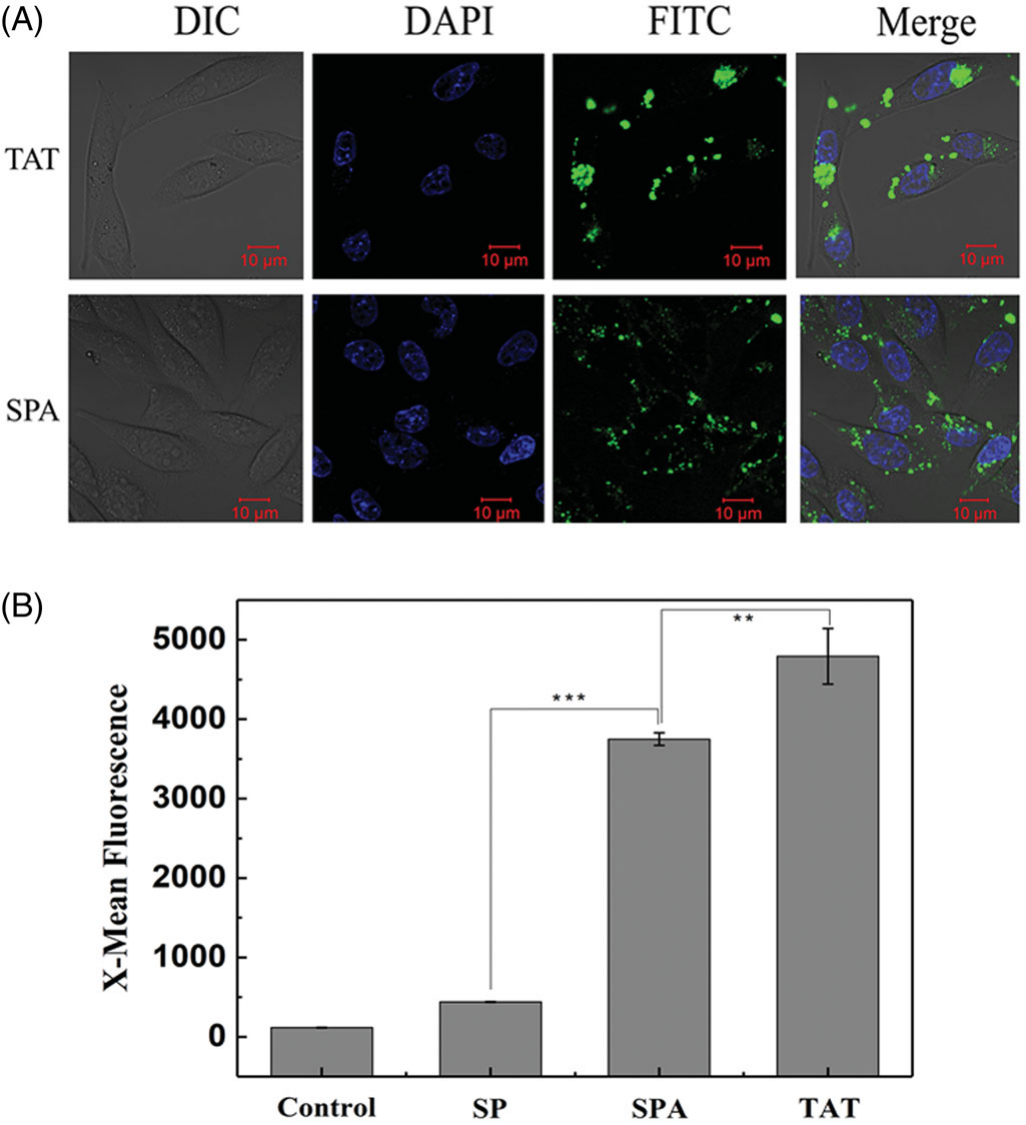
Cellular uptake of SPA in CHO cells. (A) Confocal images of SPA and TAT. Cell nuclei were counterstained with DAPI. (B) Flow cytometry assay of the cellular uptake of SP, SPA and TAT. Bar, 10 μm. **p < .01, ***p < .001.

Based on the above structural analysis, we infer that SPA can insert into the cell membrane more deeply and consequently cause higher membrane disturbance, implying that SPA possesses the capacity for cellular uptake. To determine whether SPA can enter cells, we first observed the intracellular localization of FITC-labeled SPA in CHO cells by using confocal fluorescence microscopy. As shown in Figure 1(A), confocal images indicated that SPA at 10 μM could translocate across the membranes of CHO cells and distribute inside the cells. Subsequently, we compared the uptake efficiency of SPA with those of SP and TAT at 10 μM by using flow cytometry. Figure 1(B) shows that the mean fluorescence intensity of SPA was remarkably higher than that of SP, which exhibited poor translocation into CHO cells. Unfortunately, the cellular uptake efficiency of SPA was lower than that of TAT. Nevertheless, our results confirmed that SPA indeed enters cells with relatively high efficiency.

### Cellular uptake mechanism of SPA

Despite the wide use of CPPs as delivery vectors, the cellular uptake mechanism of these peptides remains largely controversial. It is generally accepted that CPPs can enter cells via multiple pathways, including direct translocation across cell membrane and endocytosis-mediated uptake (Patel et al., 2007; Jones & Sayers, 2012; Copolovici et al., 2014; Peraro & Kritzer, 2018). However, the exact pathway through which CPPs enter cells is rarely identified because it is affected by various factors, such as cell type, physiochemical parameters of peptides, different concentrations, incubation time, cargo type and size (Jones & Sayers, 2012; Patel et al., 2007).

**Figure 2.**
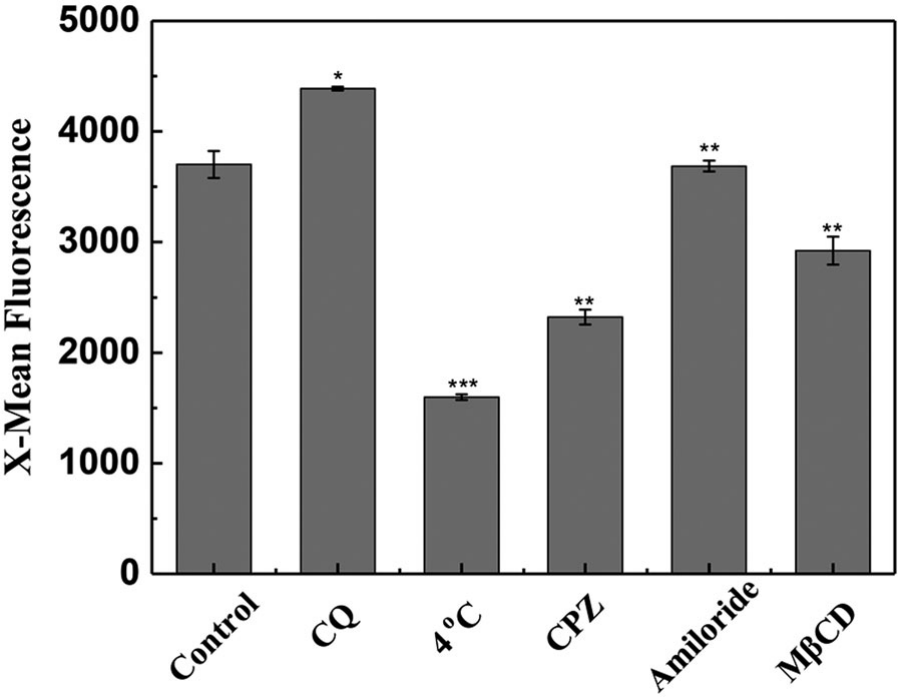
Flow cytometry assay of the cellular uptake of SPA under 4 °C, chloroquine (CQ), chlorpromazine (CPZ), amiloride and methyl-β-cyclodextrin (MβCD) treatment. The control is the fluorescence of CHO cells treated with 10 μM FITC-SPA at 37 °C for 1 h. *p < .05 versus control, **p < .01 versus control, ***p < .001 versus control.

Low temperature can suppress the cellular uptake of CPPs by the endocytosis pathway (Richard et al., 2003). In this study, the result derived from flow cytometry showed that the uptake of SPA into CHO cells was strongly suppressed at 4 °C (Figure 2), implying that the internalization of SPA involves active processes such as endocytosis. Chloroquine (CQ) is a lysosometropic agent that can promote endosomal escape of CPPs. As shown in Figure 2, cotreatment with CQ significantly increased the mean fluorescence of FITC-labeled SPA, demonstrating that a certain amount of SPA molecules were entrapped in endosomes, where acidic pH can quench fluorescein emission (Arukuusk et al., 2013; Illien et al., 2016). This result provided further evidence that SPA could enter cells by endocytosis. It is generally accepted that low concentrations of CPPs enter cells through one or more endocytosis pathways, including caveolin-mediated endocytosis, clathrin-mediated endocytosis and micropinocytosis (Patel et al., 2007). To clarify the exact endocytic pathway, we studied the specific endocytosis inhibitors on the uptake efficiency of SPA. As shown in Figure 2, our results indicated that chlorpromazine (clathrin-mediated endocytosis inhibitor) and methyl-β-cyclodextrin (caveolin-mediated endocytosis inhibitor) decreased the cellular uptake of SPA by ∼38% and 22%, respectively, while amiloride (micropinocytosis inhibitor) had no notable effect on the cellular uptake of SPA. This result suggested that SPA more likely to enter cells through clathrin- and caveolin-mediated endocytosis.

**Figure 3.**
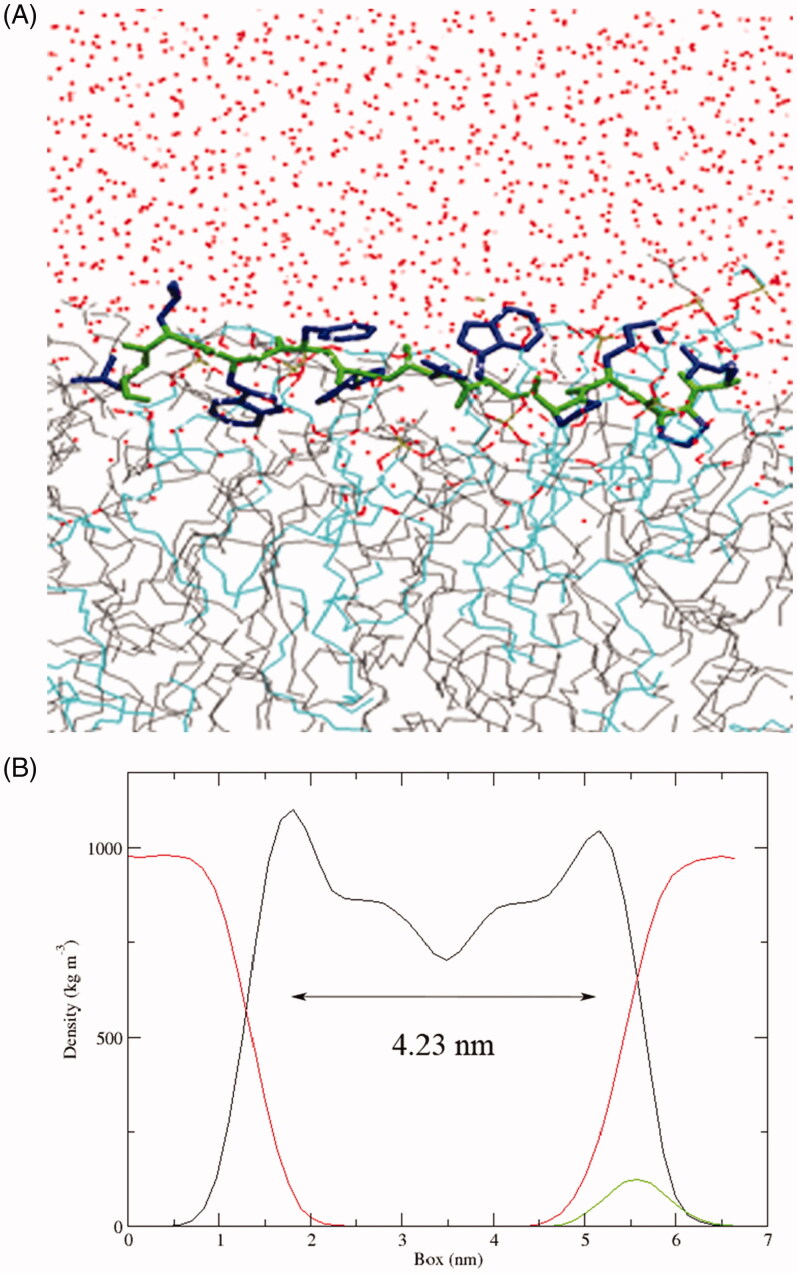
(A) Snapshots presenting the final conformations of SPA in the POPG/POPC (1:3) lipid bilayer after 200 ns simulations. The side chains of SPA are blue and the backbone is green. Water (red dots), POPC (gray line) and POPG (azure line). (B) Partial density analysis of peptide (green line), phospholipids (black line) and water (red line) in the POPG/POPC (1:3) lipid bilayer.

Although the above results demonstrated that SPA could enter cells through endocytosis-mediated uptake, we can not rule out the possibility that SPA enters cells through the direct translocation pathway. In fact, most CPPs were reported to be able to directly enter cells, especially at higher concentrations (Patel et al., 2007). Direct translocation may include different mechanisms that have been described as the formation of an inverted micelle, the nucleation of a transient pore and a carpet-like model of membrane disruption, which are similar to the action mechanisms of antimicrobial peptides (Wang et al., 2014; Guidotti et al., 2017). Our previous studies also confirmed that stearylated antimicrobial peptides [D]-K6L9 (LKLLKKLLKKLLKLL-NH_2_, the underlined letters are D-amino acids) and melittin could work as efficient CPPs for gene delivery (Zhang et al., 2013a, 2013b). SPA is similar to [D]-K6L9 in structure, which are composed of both L- and D-amino acids. In addition, our results showed that SPA could kill a variety of gram-positive and gram-negative bacteria via membrane disruption (data not shown), indicating that SPA possessed the capacity to destabilize cell membranes. Because [D]-K6L9 selectively disrupts tumor cell membranes via a carpet mechanism (Papo et al., 2004), we infer that SPA may directly translocate across cell membranes via a carpet-like mechanism: SPA accumulates parallel to the cell surface by electrostatic attraction, resulting in a thinning of the membranes followed by a small and transient membrane disruption, which permits the translocation of SPA. To obtain more details about the carpet-like mechanism of SPA, we used MD simulations to investigate the interaction of SPA with a POPG/POPC bilayer. At the end of the simulation (*t* = 200 ns), the hydrophobic D-Trp7, D-Trp9 and Leu11 inserted deeply into the lipid bilayer (Figure 3), demonstrating that these hydrophobic amino acids played an important role in the membrane insertion of SPA. In our previous work, we confirmed that the extent of membrane thinning indeed reflects the uptake efficiency of CPPs (Song et al., 2011a). The results derived from the MD simulation revealed that the membrane thickness decreased from 4.45 nm to 4.23 nm upon SPA insertion, suggesting that SPA could induce high membrane disordering. Although the MD simulation experiment did not show the whole translocation process of SPA, our results confirmed that membrane destabilization will occur after the absorption of SPA onto membranes, meaning that SPA can enter cells by directly translocating across cell membranes. Taken together, we deemed that SPA can enter cells by both endocytosis pathway and direct translocation pathway. Compared with endocytosis pathway, the merit of the direct translocation pathway is that SPA can enter cells without endosomal entrapment.

### Enzymatic stability of SPA

**Figure 4.**
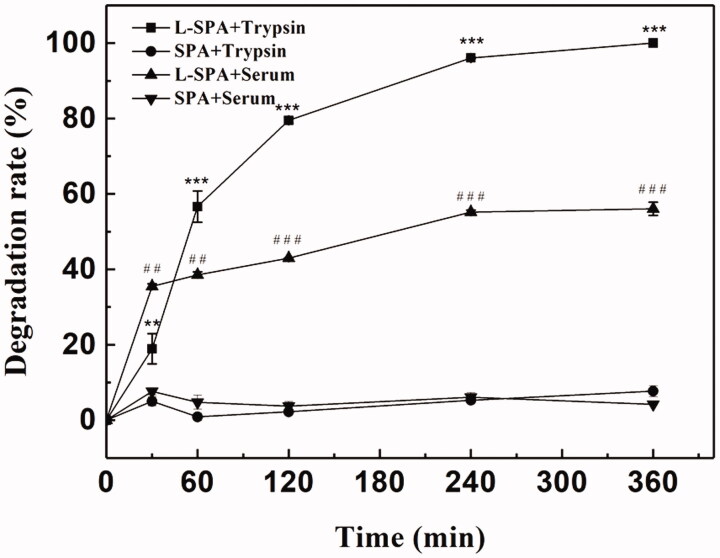
Enzymatic stability of SPA and L-SPA that consists entirely of L-amino acids against trypsin and serum. **p < .01 *vs.* SPA + trypsin, ***p < .001 *vs.* SPA + trypsin, ^##^*p* < .01 *vs.* SPA + serum, ^###^*p* < .001 *vs.* SPA + serum.

The poor stability of peptides might hinder the *in vivo* application of CPPs. Proteolytic enzymes may degrade CPPs before they reach the target sites, resulting in decreased delivery efficiency. Greater stability and uptake efficiency of CPPs can be achieved by the introduction of D-amino acids (Jiang et al., 2004; Nakase et al., 2012b; Najjar et al., 2017). As shown in Figure 4, SPA was not hydrolyzed by trypsin after incubation for 360 min, while L-SPA, which consists entirely of L-amino acids was almost completely degraded. Similarly, SPA exhibited significantly greater stability in serum than L-SPA (Figure 4). Stability in serum means that SPA can deliver more cargoes to the target sites *in vivo*.

### *In vitro* toxicity of SPA

**Figure 5.**
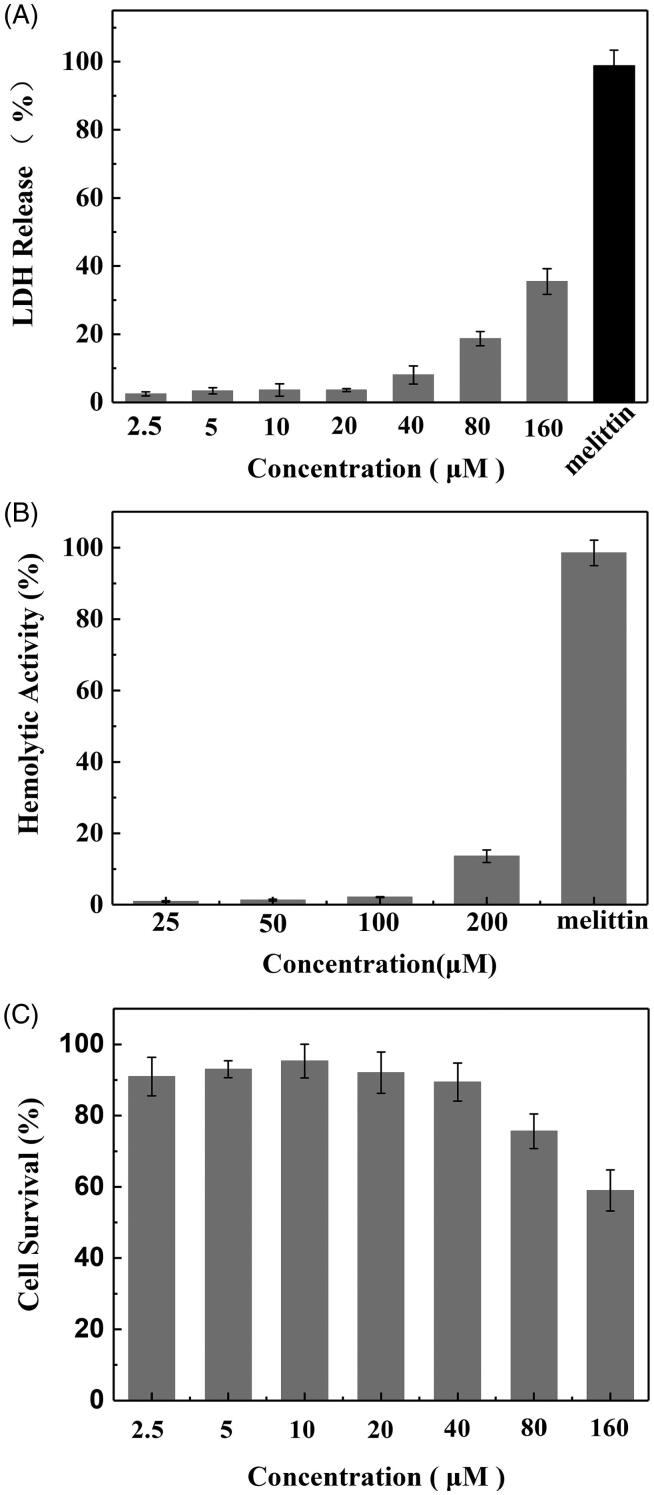
*In vitro* toxicity of SPA. (A) LDH leakage in CHO cells treated with SPA. 50 μM of melittin as positive control. (B) Hemolytic activity of SPA. 50 μM of melittin as positive control. (C) MTT assay of the viability of CHO cells treated with SPA.

Toxicity to normal cells and tissues represents a crucial barrier that would lessen the drug delivery efficiency of CPPs. To evaluate the toxic profile of SPA, the cytotoxic properties of the peptide were studied by LDH leakage, hemolysis and MTT assay. Because SPA can disrupt the cell membranes of bacteria similarly to antimicrobial peptides, we used the LDH leakage assay to measure the acute membrane disturbance of SPA. As shown in Figure 5(A), 160 μM SPA could induce only 35% LDH leakage from CHO cells, while 50 μM melittin could induce almost 100% LDH leakage. Then, we evaluated the toxicity of SPA to red blood cells. Figure 5(B) shows that SPA exhibited low hemolytic activity even at 200 μM, while the antimicrobial peptide melittin induced almost 100% lysis of red blood cells at only 50 μM. These results demonstrated that SPA exhibited relatively low membrane toxicity toward mammalian cells. The MTT assay was used to further evaluate the toxicity of SPA. Although SPA was reported to inhibit the growth of some tumor cells, SPA exhibited low cytotoxicity to CHO cells in this study (Figure 5(C)). The reason for this result is that CHO cells lack receptors on which SPA can act. In short, the above results confirmed that SPA can serve as a potent CPP with low toxicity.

### SPA-mediated CPT delivery

**Figure 6.**
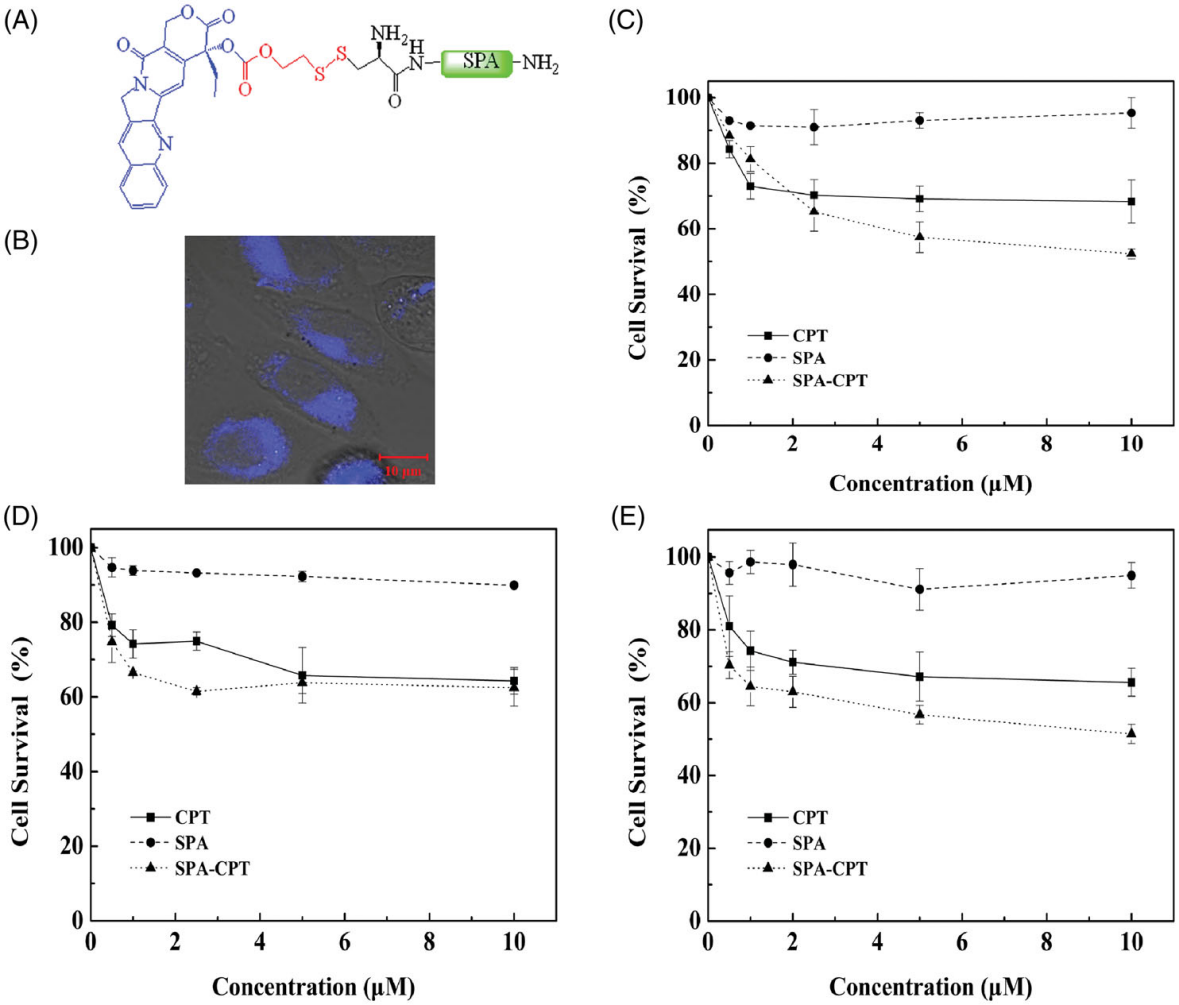
SPA-mediated camptothecin (CPT) delivery. (A) Structure of SPA-CPT conjugate. (B) Confocal images of SPA-CPT conjugate in CHO cells. Bar, 10 μm. (C) Cytotoxicity of SPA-CPT conjugate against CHO cells. (D) Cytotoxicity of SPA-CPT conjugate against MDA-MB-231 cells. (E) Cytotoxicity of SPA-CPT conjugate against U251 cells.

The attachment of traditional antitumor drugs to CPPs is an effective strategy to increase their solubility and stability, prolong drug retention, overcome drug resistance and decrease side effects, thereby improving the therapeutic efficacy of antitumor drugs (Dubikovskaya et al., 2008; Zhang et al., 2011; Dissanayake et al., 2017). Camptothecin (CPT), originally isolated from the Chinese tree Camptotheca acuminate, exerts potent antiproliferative activity against a broad range of tumors by binding to topoisomerase I [Cancer Therapies Utilizing the Camptothecins: A Reviewof the in Vivo Literature]. To evaluate the drug delivery efficiency of SPA, in this study, CPT was attached to the N-terminus of SPA by using a disulfide releasable carbonate linker (Figure 6(A)). Blue fluorescence of CPT can be observed under near UV excitation. As shown in Figure 6(B), blue fluorescence was observed in CHO cells, indicating that SPA could deliver CPT into CHO cells. Subsequently, we used the MTT assay to evaluate the cytotoxicity of the SPA-CPT conjugate against CHO cells. Figure 6(C) shows that the SPA-CPT conjugate could suppress the proliferation of CHO cells, while SPA showed no notable cytotoxicity. Compared with free CPT, SPA-CPT displayed significantly enhanced cytotoxicity against CHO cells. Furthermore, we also assessed the cytotoxicity of the SPA-CPT conjugate against MDA-MB-231 cells and U251 cells (Figure 6(D,E)). The results further demonstrated that the SPA-CPT conjugate displayed significant antitumor activity. Based on the above results, we concluded that SPA can serve as an efficient CPP with the capacity to enhance the therapeutic efficacy of traditional drugs.

### SPA-mediated gene delivery

**Figure 7.**
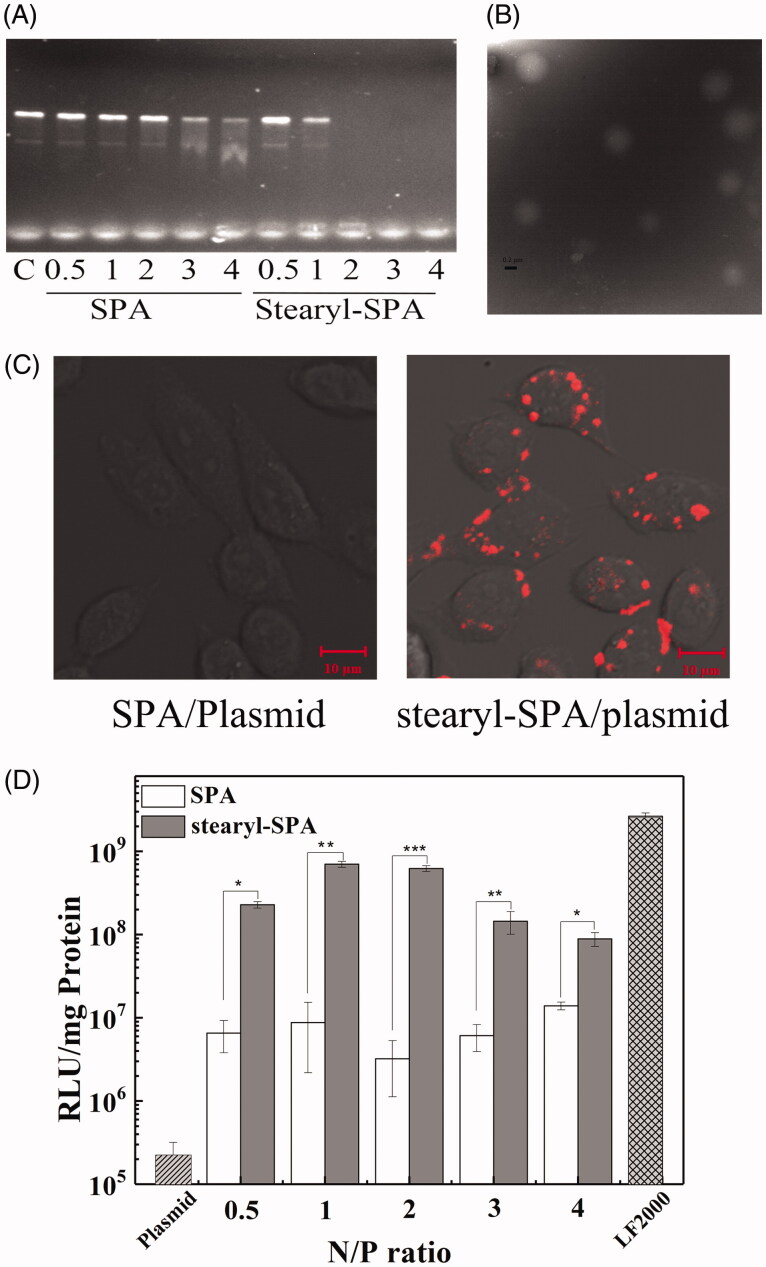
SPA-mediated gene delivery. (A) The DNA binding ability of peptides was evaluated by gel retardation assay. (B) TEM micrographs of the stearyl-SPA/pGL3 plasmids complexes at an N/P ratio of 2. Bar, 0.2 μm. (C) Confocal images of CHO cells treated with the complexes formed by stearyl-SPA with Cy5-labeled plasmids. Bar, 10 μm. (D) Transfection efficiency of SPA and stearyl-SPA in CHO cells. LF2000 was taken as a positive control. *p < .05, **p < .01, ***p < .001.

Oligonucleotide-based drugs have received considerable attention for efficiently treating heritable disorders and acquired diseases. However, nucleic acids are unable to efficiently enter cells because of strong negative charge, high molecular weight and hydrophilicity. Among the many proposed vectors, CPPs have appeared as attractive vectors for delivering nucleic acids (Boisguerin et al., 2015; Lehto et al., 2016). Stearylation has been used successfully to enhance the transfection efficiency of CPPs (Hoyer & Neundorf, 2012a, 2012b; Nakase et al., 2012a; Lehto et al., 2016). To further evaluate the application potential of SPA as an efficient CPP, we constructed a novel vector by conjugating stearic acid to the N-terminus of SPA. As shown in Figure 7(A), the DNA binding ability of stearyl-SPA peptides was significantly stronger than that of SPA, suggesting that stearylation played an important role for the DNA binding of peptides. In addition, TEM images revealed that most of the stearyl-SPA/plasmids complexes displayed a smooth and spherical shape, indicating that stearyl-SPA could efficiently condense plasmids into stable nanoparticles (Figure 7(B)). Subsequently, the results derived from confocal microscopy demonstrated that stearyl-SPA could deliver Cy5-labeled plasmids into nearly 100% of CHO cells, while unmodified SPA delivered almost no plasmids into cells (Figure 7(C)). Then, we evaluated the transfection efficiency of SPA. As shown in Figure 7(D), unmodified SPA resulted in slightly increased luciferase gene expression in CHO cells compared with that resulting from pGL3 plasmid alone. In contrast, stearyl-SPA exhibited remarkably enhanced transfection efficiency at various N/P ratios. Taken together, the above results further confirmed that SPA can serve as an efficient CPP to construct gene vectors.

## Conclusion

Compared with agonists, many antagonists have been proven to be efficient tumor-targeting molecules due to their high tumor uptake as well as antitumor activity (Sun et al., 2017). In this study, we found that the antagonist SPA could translocate into cells similarly to CPPs and efficiently deliver small hydrophobic drugs and nucleic acids into cells. In addition, our results showed that SPA exhibited low toxicity to normal cells and high stability against hydrolytic enzymes. However, we think that the cell penetrating efficiency of SPA needs to be further improved by structural optimization to enable the use of SPA as an ideal drug carrier. Nevertheless, our results demonstrated that SPA, as a more potent antagonist, can be developed as a potent CPP for efficient drug delivery. More importantly, this study opens a new avenue for designing ideal CPPs based on peptide antagonists.

## Supplementary Material

Supplemental Material

## References

[CIT0001] Araste F, Abnous K, Hashemi M, et al. (2018). Peptide-based targeted therapeutics: Focus on cancer treatment. J Control Release 292:141–62. 30408554 10.1016/j.jconrel.2018.11.004

[CIT0002] Arukuusk P, Parnaste L, Margus H, et al. (2013). Differential endosomal pathways for radically modified peptide vectors. Bioconjugate Chem 24:1721–32. 10.1021/bc4002757 23981119

[CIT0003] Boisguerin P, Deshayes S, Gait MJ, et al. (2015). Delivery of therapeutic oligonucleotides with cell penetrating peptides. Adv Drug Deliv Rev 87:52–67. 25747758 10.1016/j.addr.2015.02.008 PMC7102600

[CIT0004] Cescato R, Maina T, Nock B, et al. (2008). Bombesin receptor antagonists may be preferable to agonists for tumor targeting. J Nucl Med 49:318–26. 18199616 10.2967/jnumed.107.045054

[CIT0005] Copolovici DM, Langel K, Eriste E, et al. (2014). Cell-Penetrating peptides: design, synthesis, and applications. Acs Nano 8:1972–94. 24559246 10.1021/nn4057269

[CIT0006] Datar P, Srivastava S, Coutinho E, et al. (2004). Substance P: Structure, function, and therapeutics. Curr Top Med Chem 4:75–103. 14754378 10.2174/1568026043451636

[CIT0007] Dissanayake S, Denny WA, Gamage S, et al. (2017). Recent developments in anticancer drug delivery using cell penetrating and tumor targeting peptides. J Control Release 250:62–76. 28167286 10.1016/j.jconrel.2017.02.006

[CIT0008] Dubikovskaya EA, Thorne SH, Pillow TH, et al. (2008). Overcoming multidrug resistance of small-molecule therapeutics through conjugation with releasable octaarginine transporters. Proc Natl Acad Sci USA 105:12128–33. 18713866 10.1073/pnas.0805374105 PMC2527877

[CIT0009] Ginj M, Zhang HW, Waser B, et al. (2006). Radiolabeled somatostatin receptor antagonists are preferable to agonists for *in vivo* peptide receptor targeting of tumors. Proc Natl Acad Sci USA 103:16436–41. 17056720 10.1073/pnas.0607761103 PMC1618814

[CIT0010] Guha S, Eibl G, Kisfalvi K, et al. (2005). Broad-spectrum G protein-coupled receptor antagonist, [D-Arg(1) D-Trp(5,7,9),Leu(11)]SP: A dual inhibitor of growth and angiogenesis in pancreatic cancer. Cancer Res 65:2738–45. 15805273 10.1158/0008-5472.CAN-04-3197

[CIT0011] Guidotti G, Brambilla L, Rossi D. (2017). Cell-penetrating peptides: From basic research to clinics. Trends Pharmacol Sci 38:406–24. 28209404 10.1016/j.tips.2017.01.003

[CIT0012] Gupta A, Mandal D, Ahmadibeni Y, et al. (2011). Hydrophobicity drives the cellular uptake of short cationic peptide ligands. Eur Biophys J 40:727–36. 21409455 10.1007/s00249-011-0685-4

[CIT0013] Henne WA, Doorneweerd DD, Hilgenbrink AR, et al. (2006). Synthesis and activity of a folate peptide camptothecin prodrug. Bioorg Med Chem Lett 16:5350–5. 16901694 10.1016/j.bmcl.2006.07.076

[CIT0014] Hoyer J, Neundorf I. (2012a). Knockdown of a G protein-coupled receptor through efficient peptide-mediated siRNA delivery. J Control Release 161:826–34. 22626516 10.1016/j.jconrel.2012.05.017

[CIT0015] Hoyer J, Neundorf I. (2012b). Peptide vectors for the nonviral delivery of nucleic acids. Acc Chem Res 45:1048–56. 22455499 10.1021/ar2002304

[CIT0016] Illien F, Rodriguez N, Amoura M, et al. (2016). Quantitative fluorescence spectroscopy and flow cytometry analyses of cell-penetrating peptides internalization pathways: optimization, pitfalls, comparison with mass spectrometry quantification. Sci Rep 6:36938. 27841303 10.1038/srep36938 PMC5107916

[CIT0017] Jarver P, Mager I, Langel U. (2010). In vivo biodistribution and efficacy of peptide mediated delivery. Trends Pharmacol Sci 31:528–35. 20828841 10.1016/j.tips.2010.07.006

[CIT0018] Jiang T, Olson ES, Nguyen QT, et al. (2004). Tumor imaging by means of proteolytic activation of cell-penetrating peptides. Proc Natl Acad Sci USA 101:17867–72. 15601762 10.1073/pnas.0408191101 PMC539314

[CIT0019] Jobin ML, Blanchet M, Henry S, et al. (2015). The role of tryptophans on the cellular uptake and membrane interaction of arginine-rich cell penetrating peptides. Biochim Biophys Acta 1848:593–602. 25445669 10.1016/j.bbamem.2014.11.013

[CIT0020] Jones AT, Sayers EJ. (2012). Cell entry of cell penetrating peptides: tales of tails wagging dogs. J Control Release 161:582–91. 22516088 10.1016/j.jconrel.2012.04.003

[CIT0021] Kabelka I, Vacha R. (2018). Optimal hydrophobicity and reorientation of amphiphilic peptides translocating through membrane. Biophys J 115:1045–54. 30177443 10.1016/j.bpj.2018.08.012 PMC6139821

[CIT0022] Kauffman WB, Fuselier T, He J, et al. (2015). Mechanism matters: a taxonomy of cell penetrating peptides. Trends Biochem Sci 40:749–64. 26545486 10.1016/j.tibs.2015.10.004 PMC4727446

[CIT0023] Kim S, Kim SS, Lee BJ. (2005). Correlation between the activities of alpha-helical antimicrobial peptides and hydrophobicities represented as RP HPLC retention times. Peptides 26:2050–6. 15894405 10.1016/j.peptides.2005.04.007

[CIT0024] Komin A, Russell LM, Hristova KA, et al. (2017). Peptide-based strategies for enhanced cell uptake, transcellular transport, and circulation: Mechanisms and challenges. Adv Drug Deliv Rev 110-111:52–64. 27313077 10.1016/j.addr.2016.06.002

[CIT0025] Koren E, Torchilin VP. (2012). Cell-penetrating peptides: breaking through to the other side. Trends Mol Med 18:385–93. 22682515 10.1016/j.molmed.2012.04.012

[CIT0026] Lehto T, Ezzat K, Wood MJA, et al. (2016). Peptides for nucleic acid delivery. Adv Drug Deliv Rev 106:172–82. 27349594 10.1016/j.addr.2016.06.008

[CIT0027] Majumdar S, Siahaan TJ. (2012). Peptide-mediated targeted drug delivery. Med Res Rev 32:637–58. 20814957 10.1002/med.20225

[CIT0028] Mickan A, Sarko D, Haberkorn U, et al. (2014). Rational design of CPP-based drug delivery systems: considerations from pharmacokinetics. Curr Pharm Biotechnol 15:200–9. 25312539 10.2174/138920101503140822101814

[CIT0029] Milletti F. (2012). Cell-penetrating peptides: classes, origin, and current landscape. Drug Discov Today 17:850–60. 22465171 10.1016/j.drudis.2012.03.002

[CIT0030] Najjar K, Erazo-Oliveras A, Brock DJ, et al. (2017). An l- to d-Amino acid conversion in an endosomolytic analog of the Cell-penetrating peptide TAT influences proteolytic stability, endocytic uptake, and endosomal escape. J Biol Chem 292:847–61. 27923812 10.1074/jbc.M116.759837 PMC5247658

[CIT0031] Nakase I, Akita H, Kogure K, et al. (2012a). Efficient intracellular delivery of nucleic acid pharmaceuticals using cell-penetrating peptides. Acc Chem Res 45:1132–9. 22208383 10.1021/ar200256e

[CIT0032] Nakase I, Konishi Y, Ueda M, et al. (2012b). Accumulation of arginine-rich cell-penetrating peptides in tumors and the potential for anticancer drug delivery in vivo. J Control Release 159:181–8. 22285548 10.1016/j.jconrel.2012.01.016

[CIT0033] Papo N, Braunstein A, Eshhar Z, et al. (2004). Suppression of human prostate tumor growth in mice by a cytolytic D-, L-amino Acid Peptide: membrane lysis, increased necrosis, and inhibition of prostate-specific antigen secretion. Cancer Res 64:5779–86. 15313920 10.1158/0008-5472.CAN-04-1438

[CIT0034] Patel LN, Zaro JL, Shen WC. (2007). Cell penetrating peptides: intracellular pathways and pharmaceutical perspectives. Pharm Res 24:1977–92. 17443399 10.1007/s11095-007-9303-7

[CIT0035] Peraro L, Kritzer JA. (2018). Emerging methods and design principles for cell-penetrant peptides. Angew Chem Int Ed Engl 57:11868–81. 29740917 10.1002/anie.201801361 PMC7184558

[CIT0036] Richard JP, Melikov K, Vives E, et al. (2003). Cell-penetrating peptides - A reevaluation of the mechanism of cellular uptake. J Biol Chem 278:585–90. 12411431 10.1074/jbc.M209548200

[CIT0037] Rydberg HA, Matson M, Amand HL, et al. (2012). Effects of tryptophan content and backbone spacing on the uptake efficiency of cell-penetrating peptides. Biochemistry 51:5531–9. 22712882 10.1021/bi300454k

[CIT0038] Seckl MJ, Higgins T, Widmer F, et al. (1997). [D-Arg(1),D-Trp(5,7,9),Leu(11)]Substance P: A novel potent inhibitor of signal transduction and growth *in vitro* and in vivo in small cell lung cancer cells. Cancer Res 57:51–4. 8988040

[CIT0039] Song J, Kai M, Zhang W, et al. (2011a). Cellular uptake of transportan 10 and its analogs in live cells: Selectivity and structure-activity relationship studies. Peptides 32:1934–41. 21827806 10.1016/j.peptides.2011.07.018

[CIT0040] Song JJ, Kai M, Zhang W, et al. (2011b). Cellular uptake of transportan 10 and its analogs in live cells: Selectivity and structure-activity relationship studies. Peptides 32:1934–41. 21827806 10.1016/j.peptides.2011.07.018

[CIT0041] Song JJ, Zhang W, Kai M, et al. (2013). Design of an acid-activated antimicrobial peptide for tumor therapy. Mol Pharmaceutics 10:2934–41. 10.1021/mp400052s 23819484

[CIT0042] Song JJ, Zhang Y, Zhang W, et al. (2015). Cell penetrating peptide TAT can kill cancer cells via membrane disruption after attachment of camptothecin. Peptides 63:143–9. 25496911 10.1016/j.peptides.2014.12.001

[CIT0043] Steinhoff MS, von Mentzer B, Geppetti P, et al. (2014). Tachykinins and their receptors: contributions to physiological control and the mechanisms of disease. Physiol Rev 94:265–301. 24382888 10.1152/physrev.00031.2013 PMC3929113

[CIT0044] Sun XL, Li YS, Liu T, et al. (2017). Peptide-based imaging agents for cancer detection. Adv Drug Deliv Rev 110:38–51. 27327937 10.1016/j.addr.2016.06.007 PMC5235994

[CIT0045] Svensen N, Walton JG, Bradley M. (2012). Peptides for cell-selective drug delivery. Trends Pharmacol Sci 33:186–92. 22424670 10.1016/j.tips.2012.02.002

[CIT0046] Wang FH, Wang Y, Zhang X, et al. (2014). Recent progress of cell-penetrating peptides as new carriers for intracellular cargo delivery. J Control Release 174:126–36. 24291335 10.1016/j.jconrel.2013.11.020

[CIT0047] Yau WM, Wimley WC, Gawrisch K, et al. (1998). The preference of tryptophan for membrane interfaces. Biochemistry 37:14713–8. 9778346 10.1021/bi980809c

[CIT0048] Zhang W, Song J, Liang R, et al. (2013a). Stearylated antimicrobial peptide [D]-K6L9 with cell penetrating property for efficient gene transfer. Peptides 46:33–9. 23727033 10.1016/j.peptides.2013.05.011

[CIT0049] Zhang W, Song J, Liang R, et al. (2013b). Stearylated antimicrobial peptide melittin and its retro isomer for efficient gene transfection. Bioconjugate Chem 24:1805–12. 10.1021/bc400053b 24107137

[CIT0050] Zhang W, Song JJ, Zhang BZ, et al. (2011). Design of acid-activated cell penetrating peptide for delivery of active molecules into cancer cells. Bioconjugate Chem 22:1410–5. 10.1021/bc200138d 21663318

