# Supplementary material for: SPA: a peptide antagonist that acts as a cell-penetrating peptide for drug delivery
Source: Drug Deliv. 2019 Dec 24;27(1):91–9. doi: 10.1080/10717544.2019.1706669 (PMC6968712; doi:10.1080/10717544.2019.1706669)

Table S1

| Peptide | Sequence | M_cal_^a^ | M_obs_^b^ | retention  time (min) | Purity |
| --- | --- | --- | --- | --- | --- |
| SPA | rPKPwQwFwLL-NH_2_ | 1555.9 | 1557.8 | 21.0 | 97.4% |
| FITC-SPA | FITC-AHX-rPKPwQwFwLL-NH_2_ | 2059.1 | 2060.0 | 21.2 | 97.9% |
| Stearyl-SPA | C18-rPKPwQwFwLL-NH_2_ | 1822.4 | 1822.1 | 29.9 | 97% |
| L-SPA | RPKPWQWFWLL-NH_2_ | 1555.9 | 1555.9 | 18.6 | 98.8 |
| SP | RPKPQQFFGLM-NH_2_ | 1347.8 | 1347.6 | 16.2 | 97.3% |
| FITC-SP | FITC-AHX-RPKPQQFFGLM-NH_2_ | 1850.3 | 1849.8 | 17.2 | 95.3% |
| FITC-TAT | FITC-AHX-YGRKKRRQRRR-NH_2_ | 2061.4 | 2063.4 | 16.0 | 97.1% |
| melittin | GIGAVLKVLTTGLPALISWIKRKRQQ-NH_2_ | 2846.8 | 2845.7 | 18.8 | 96% |
| SPA-CPT | CPT-rPKPwQwFwLL-NH_2_ | 2109.5 | 2109.4 | 21.8 | 98.5% |
| ^a^Calculated monoisotopic masses.  ^b^Observed monoisotopic masses.  AHX: aminocaproic acid | | | | | |

Figure S1:

SPA:

HPLC:


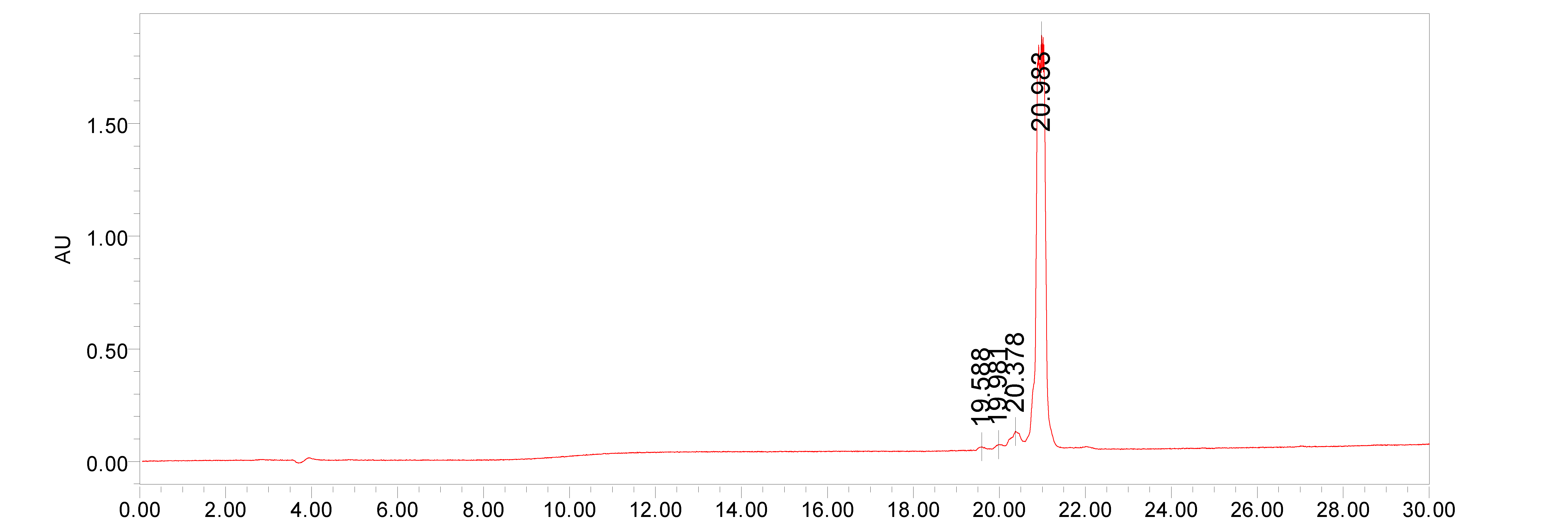


MS:


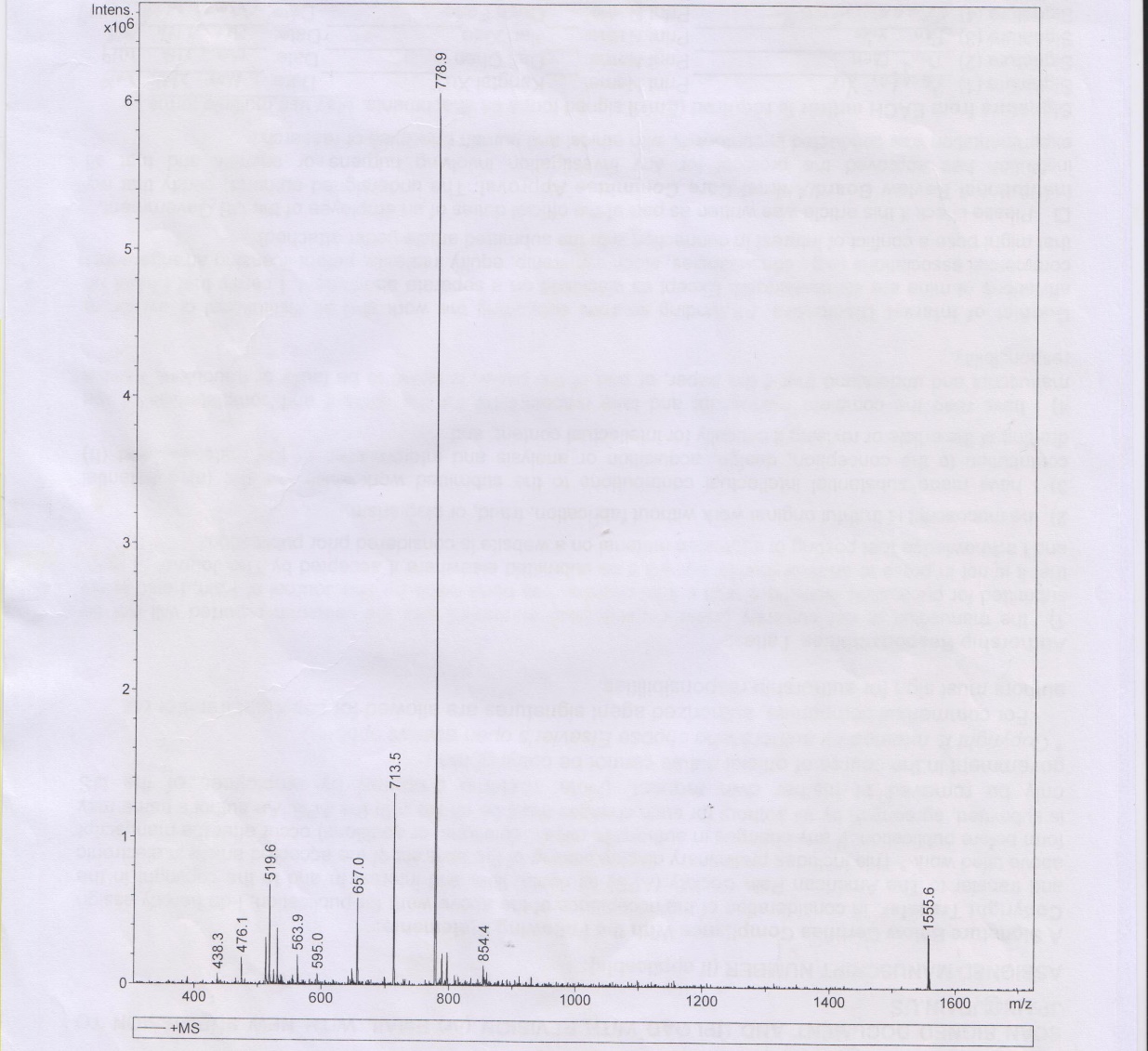


Figure S2:

FITC-SPA

HPLC:


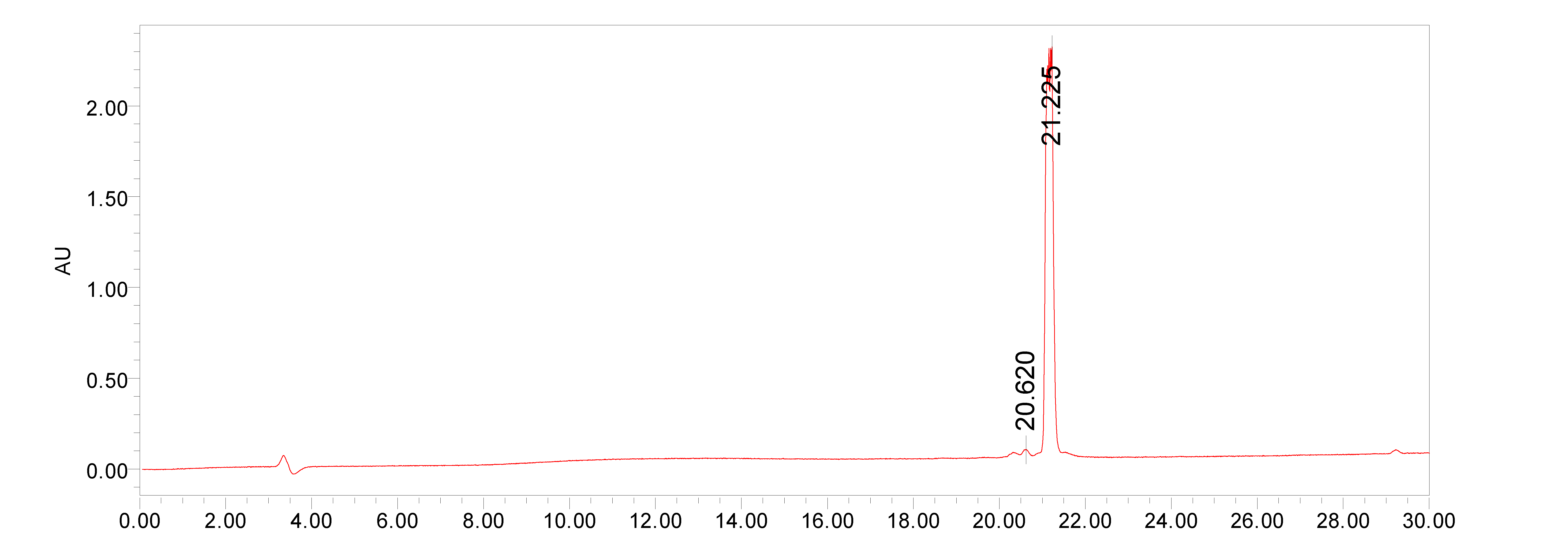


MS:


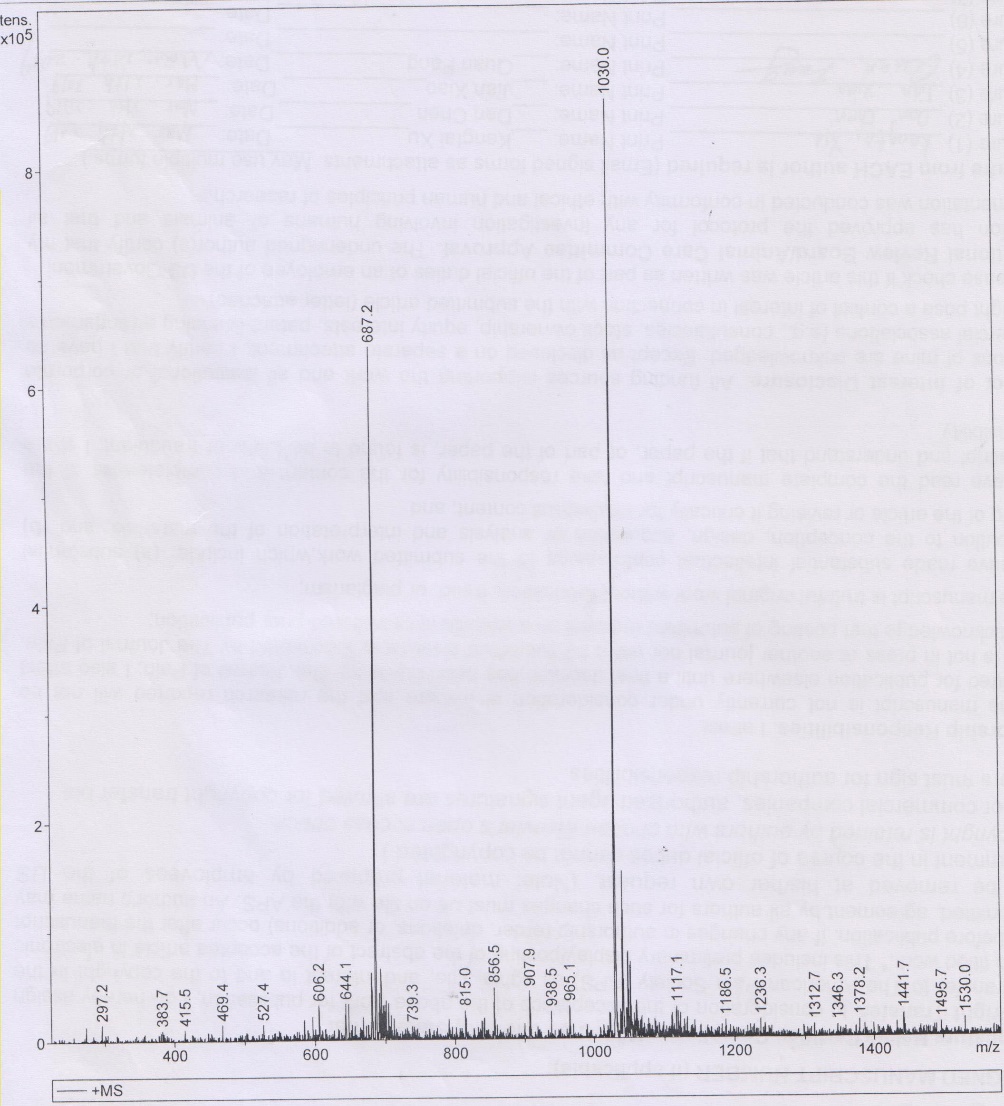


Figure S3:

Stearyl-SPA

HPLC:


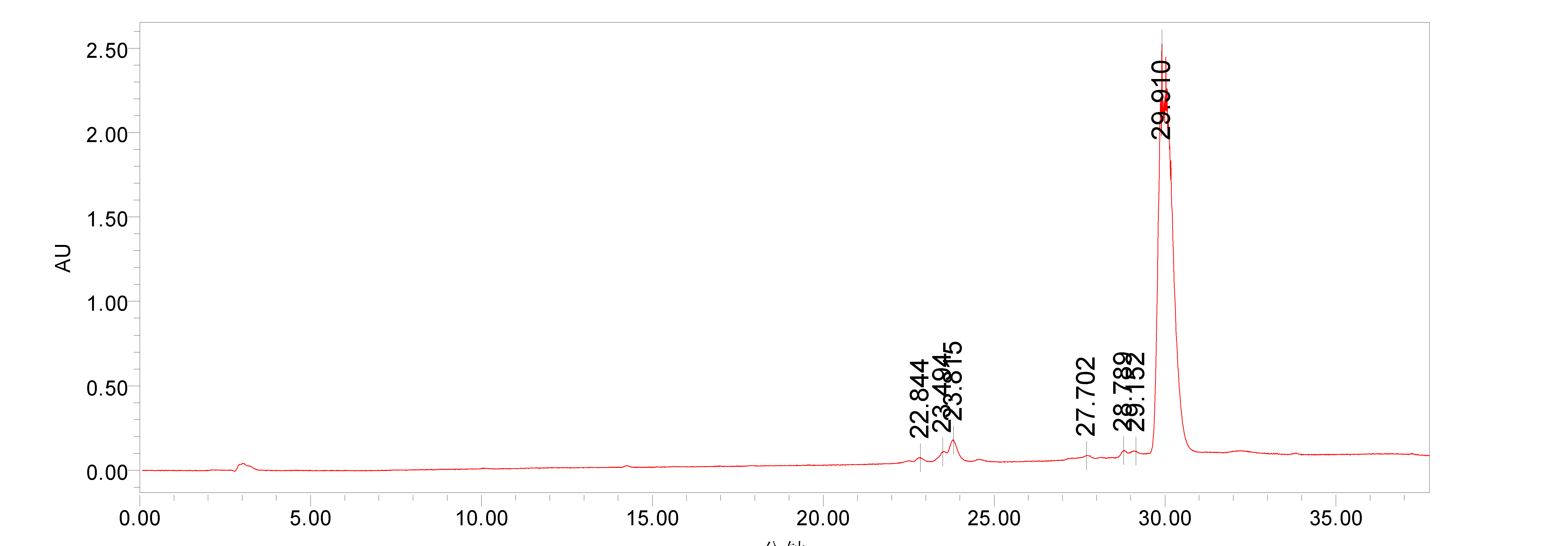


MS:


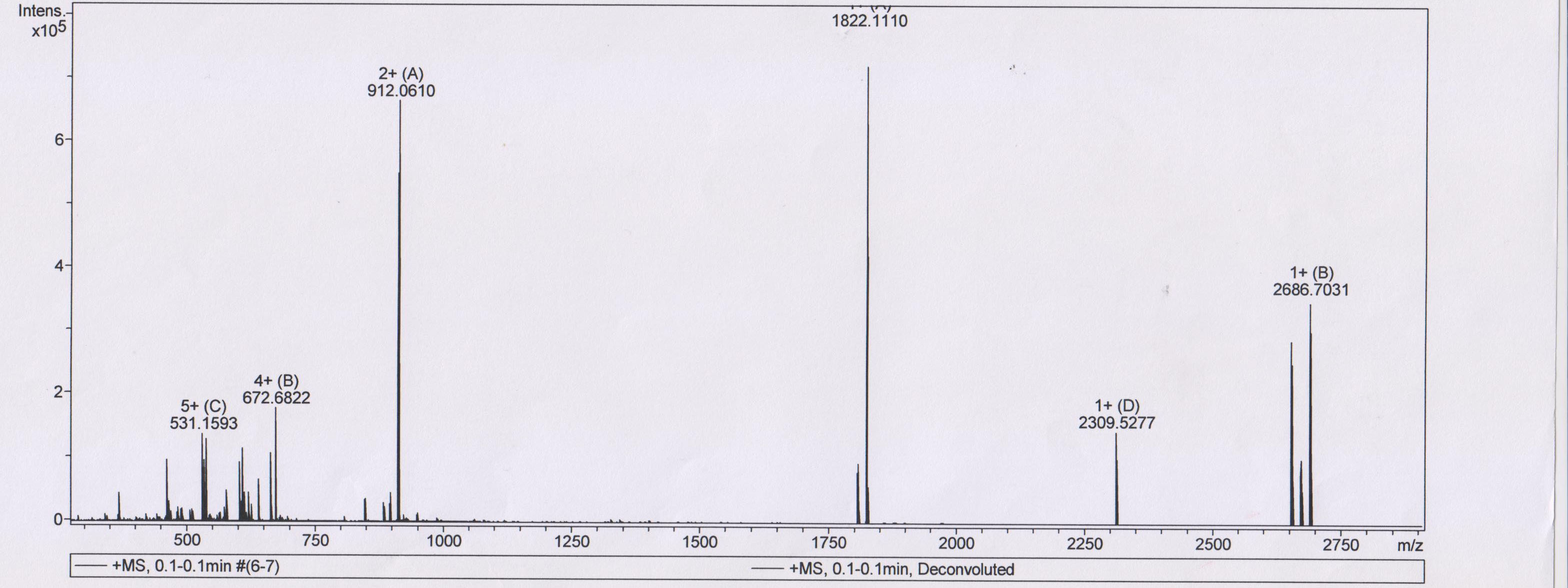


Figure S4:

L-SPA

HPLC:


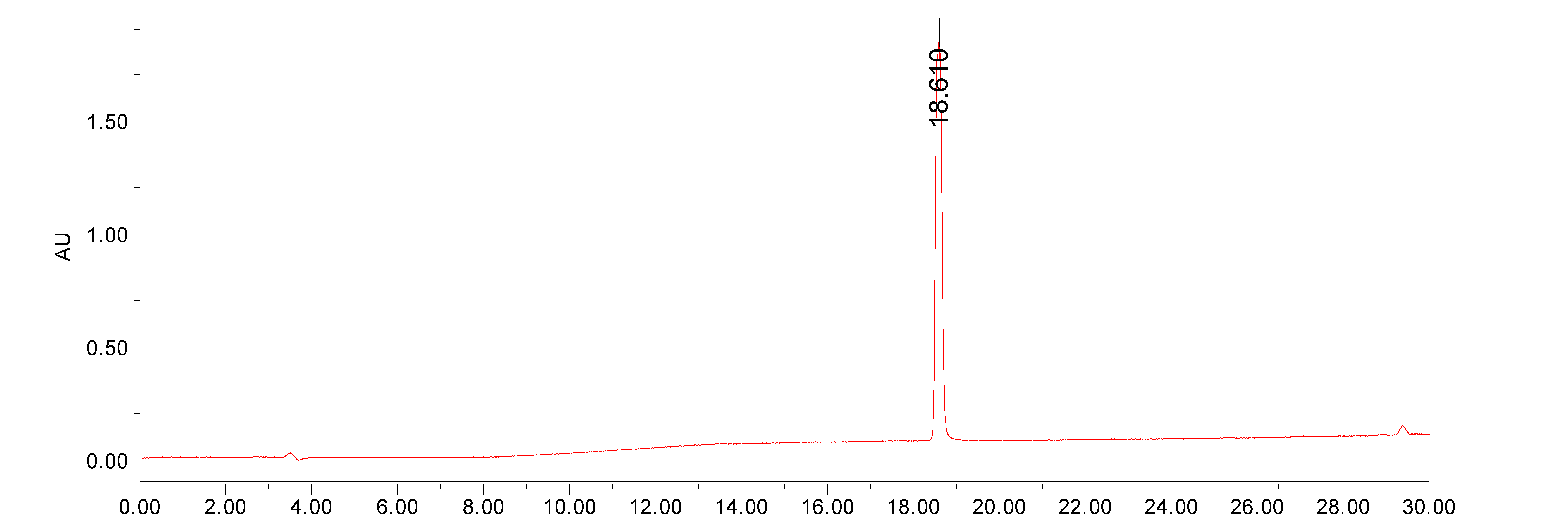


MS:


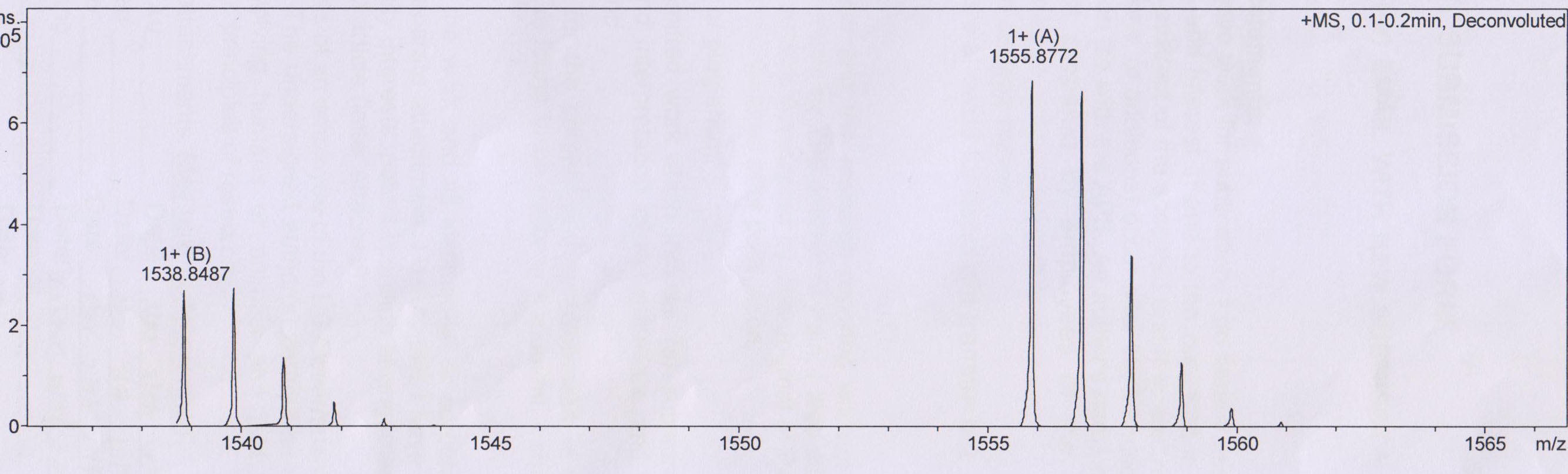


Figure S5:

SP

HPLC:


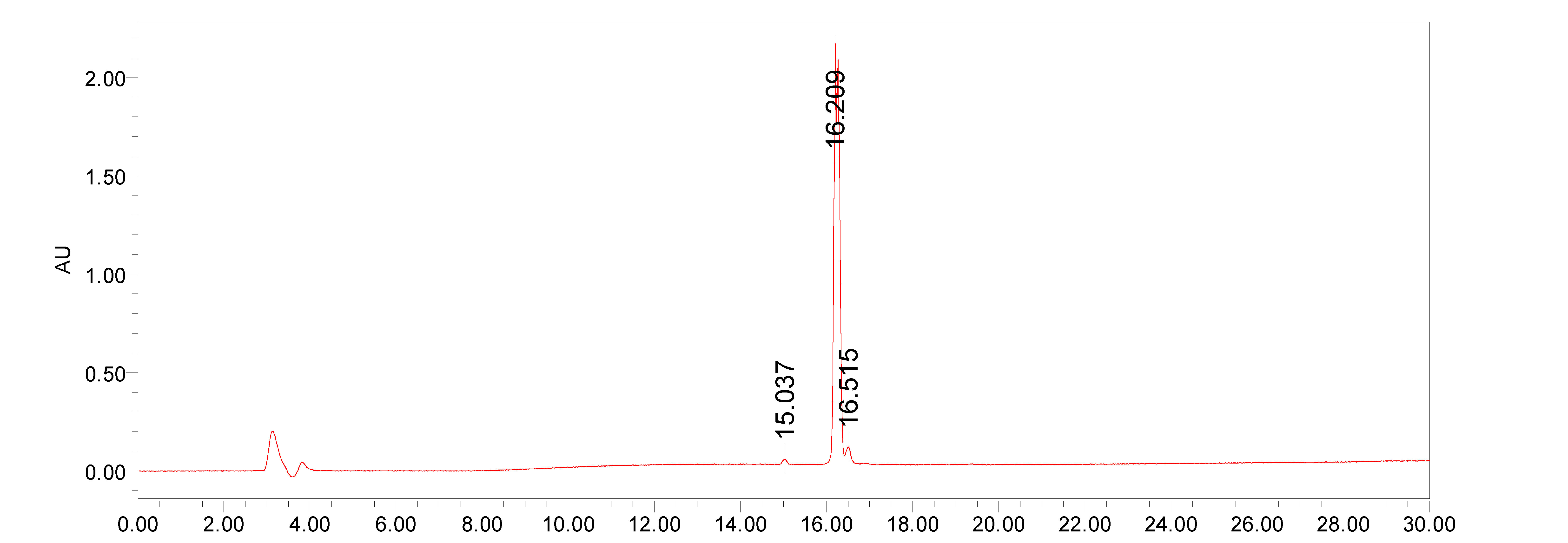


MS:


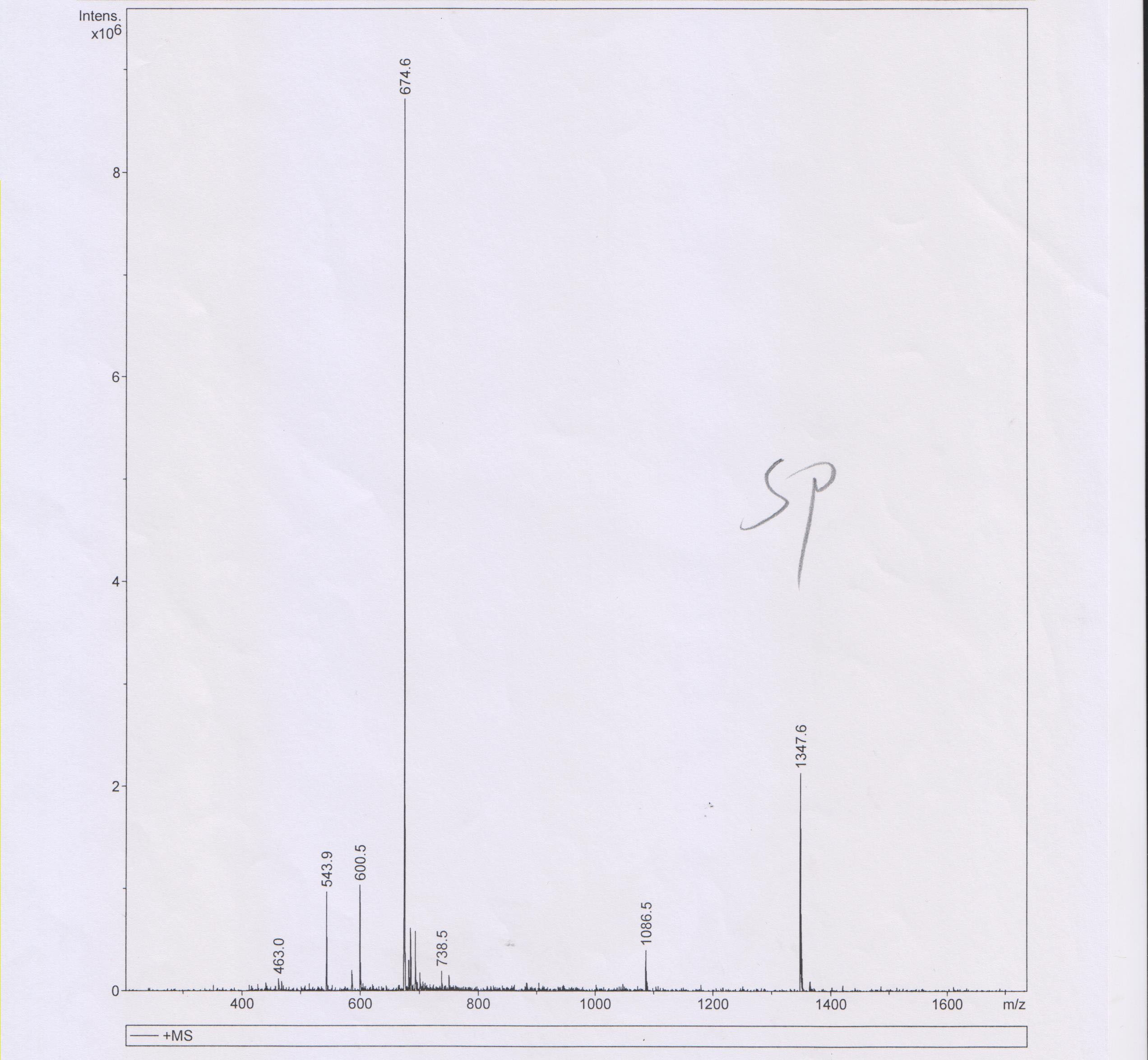


Figure S6:

FITC-SP

HPLC:


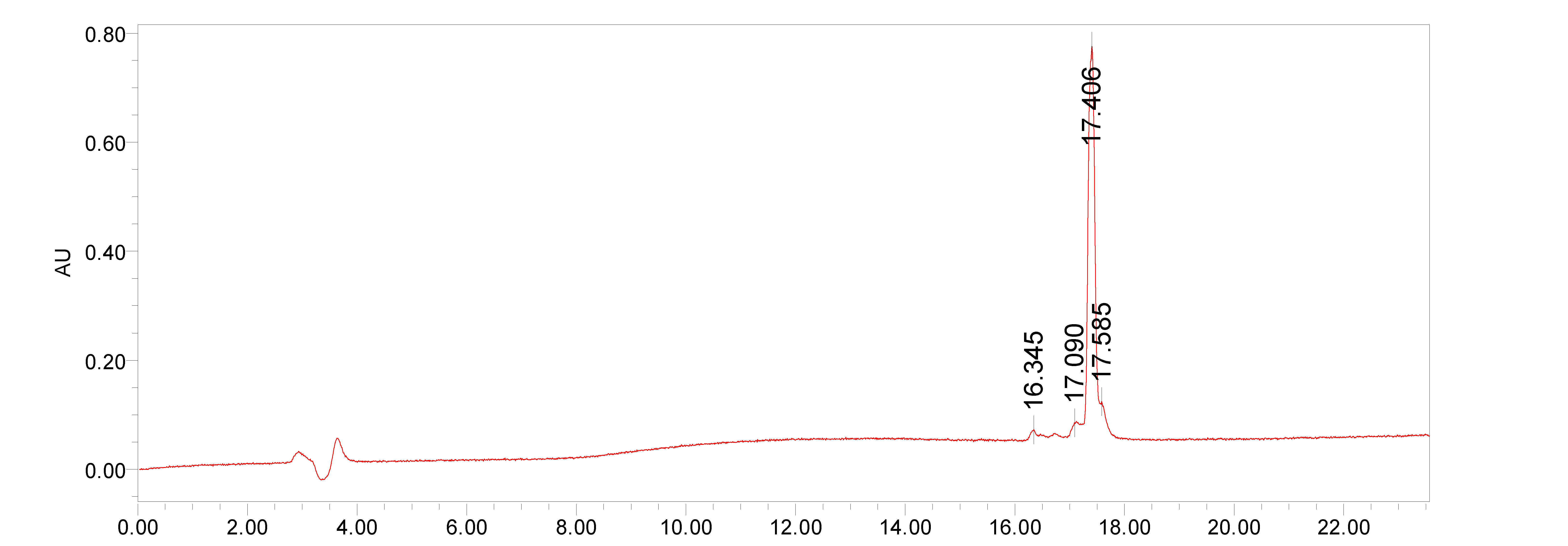


MS:


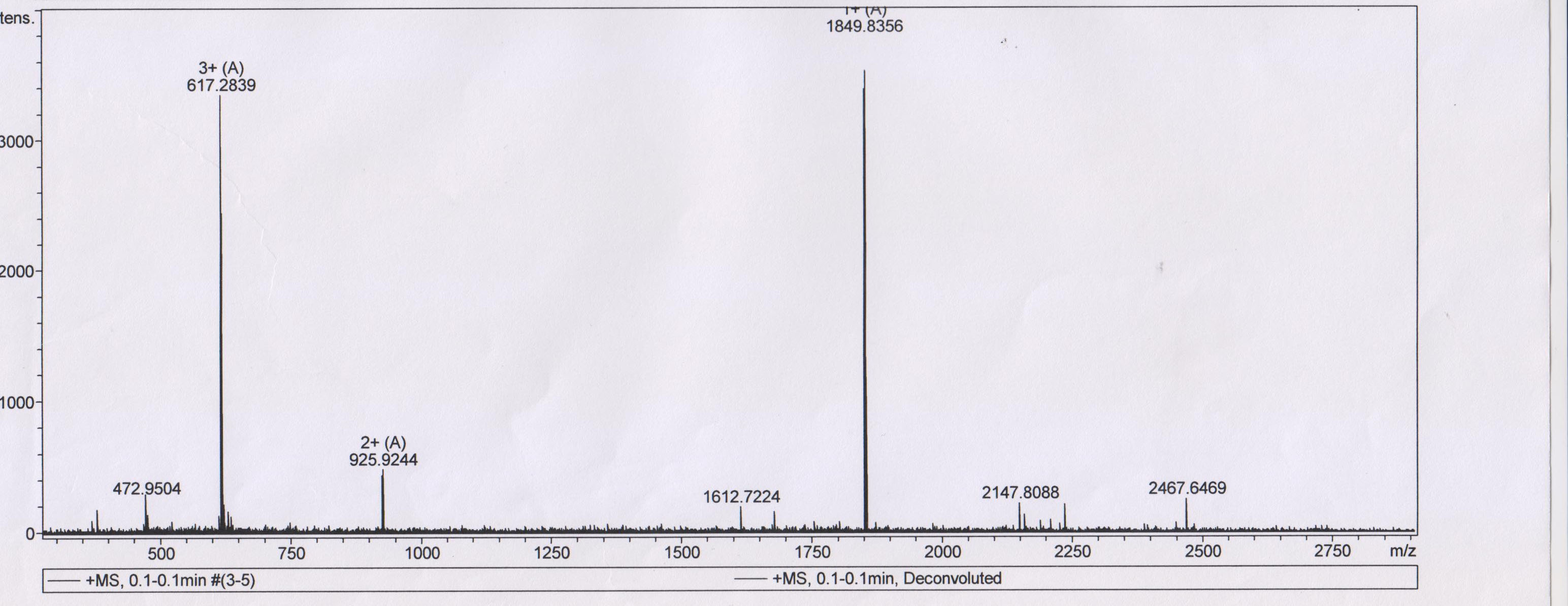


Figure S7:

FITC-TAT

HPLC:


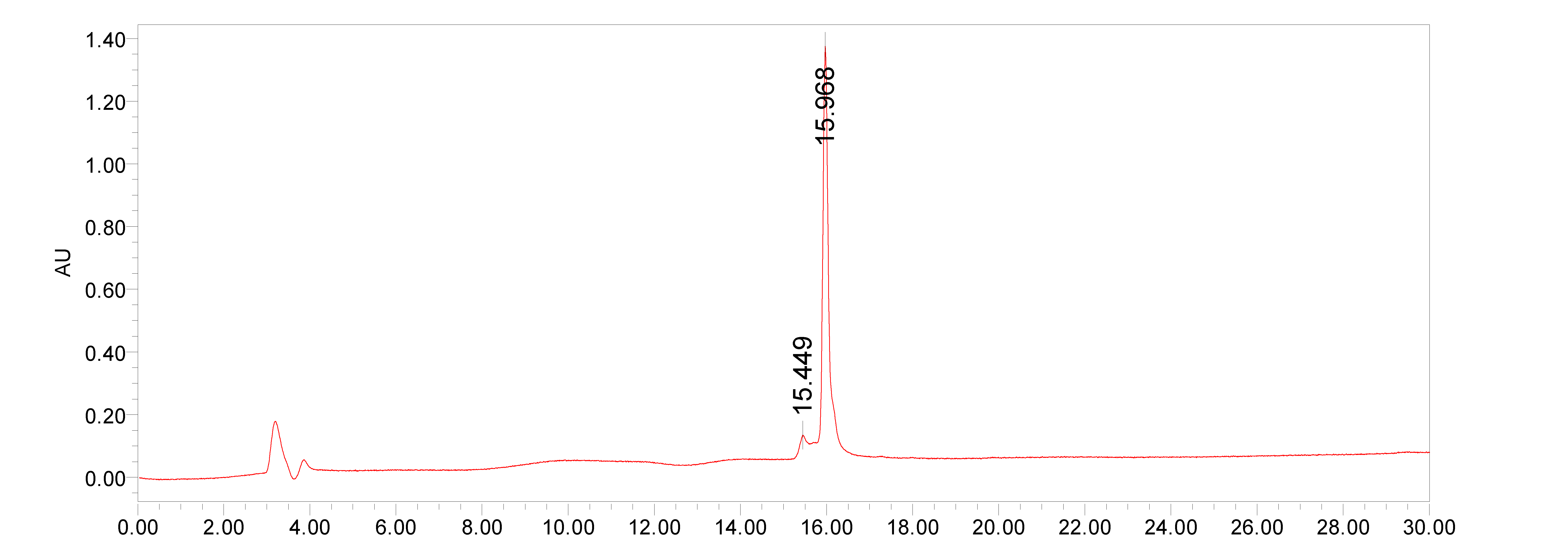


MS:


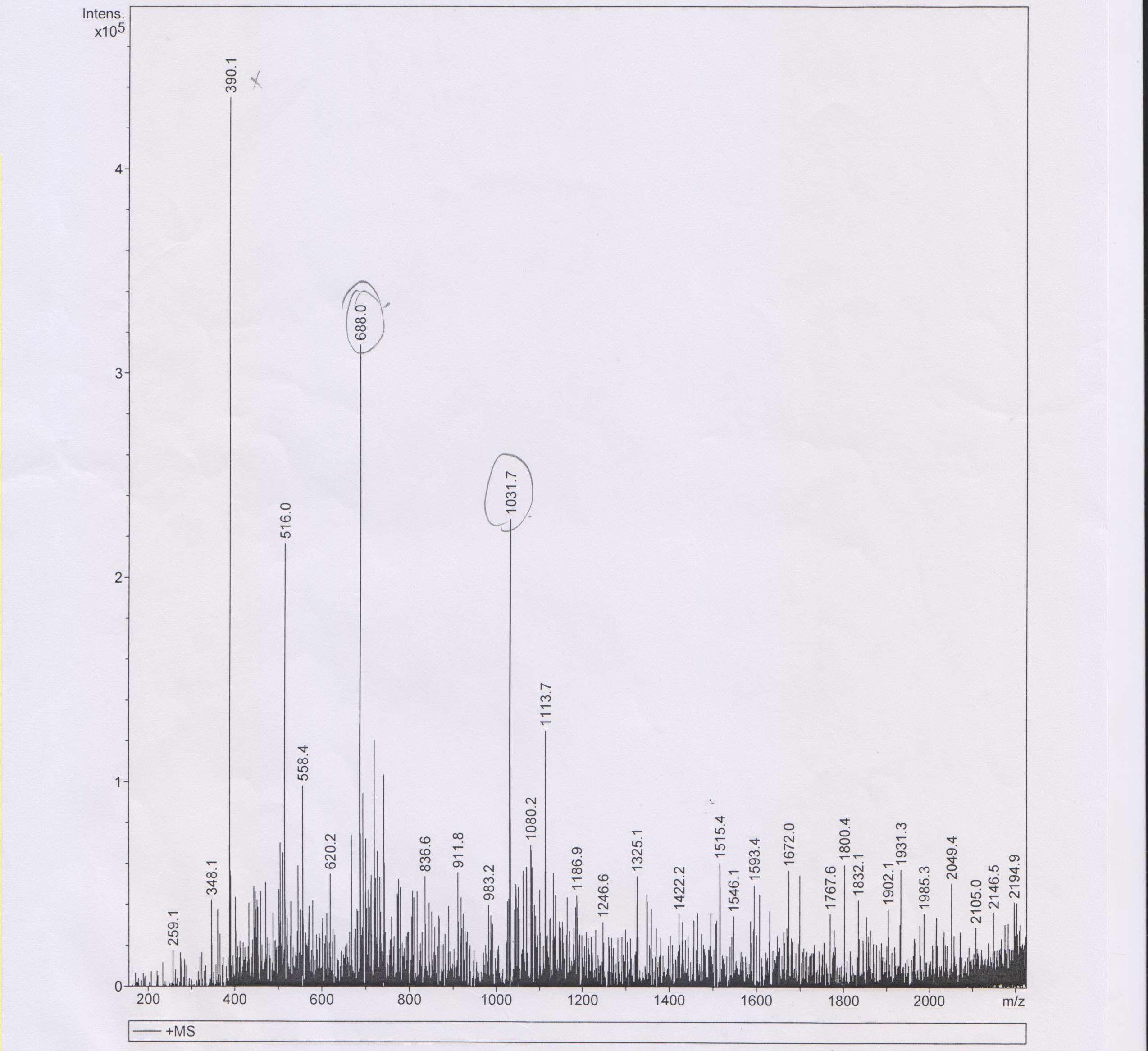


Figure S8:

melittin

HPLC:


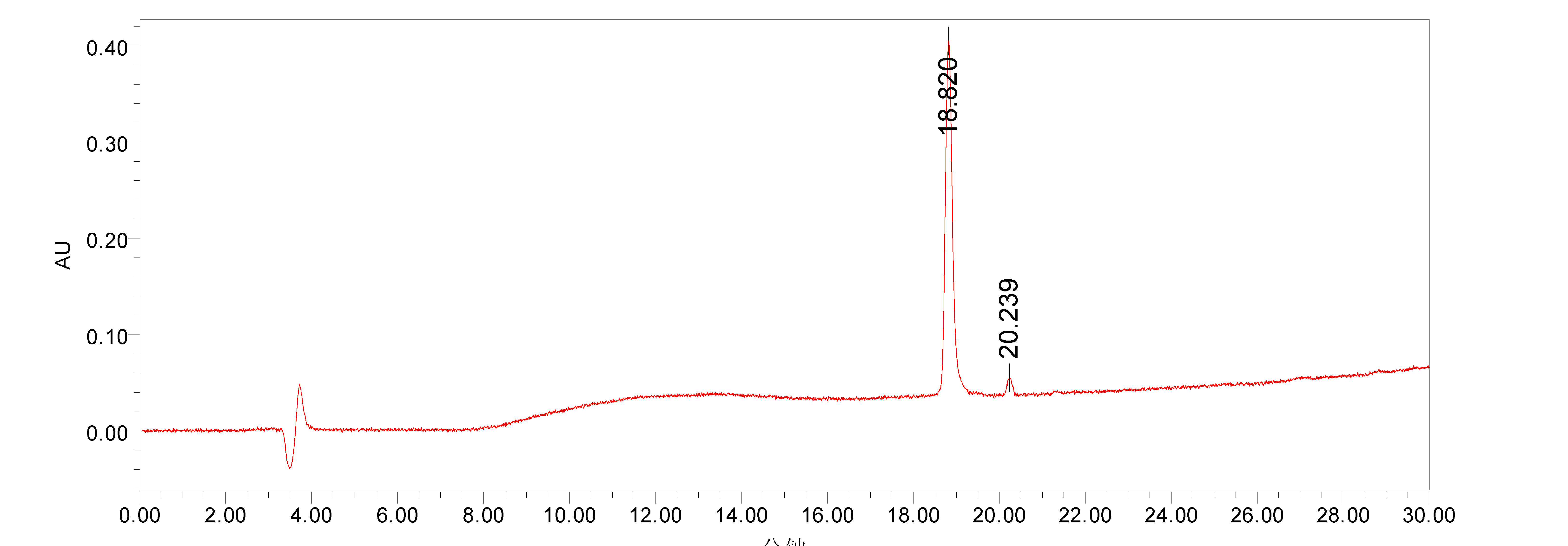


MS:





Figure S9:

SPA-CPT

HPLC:


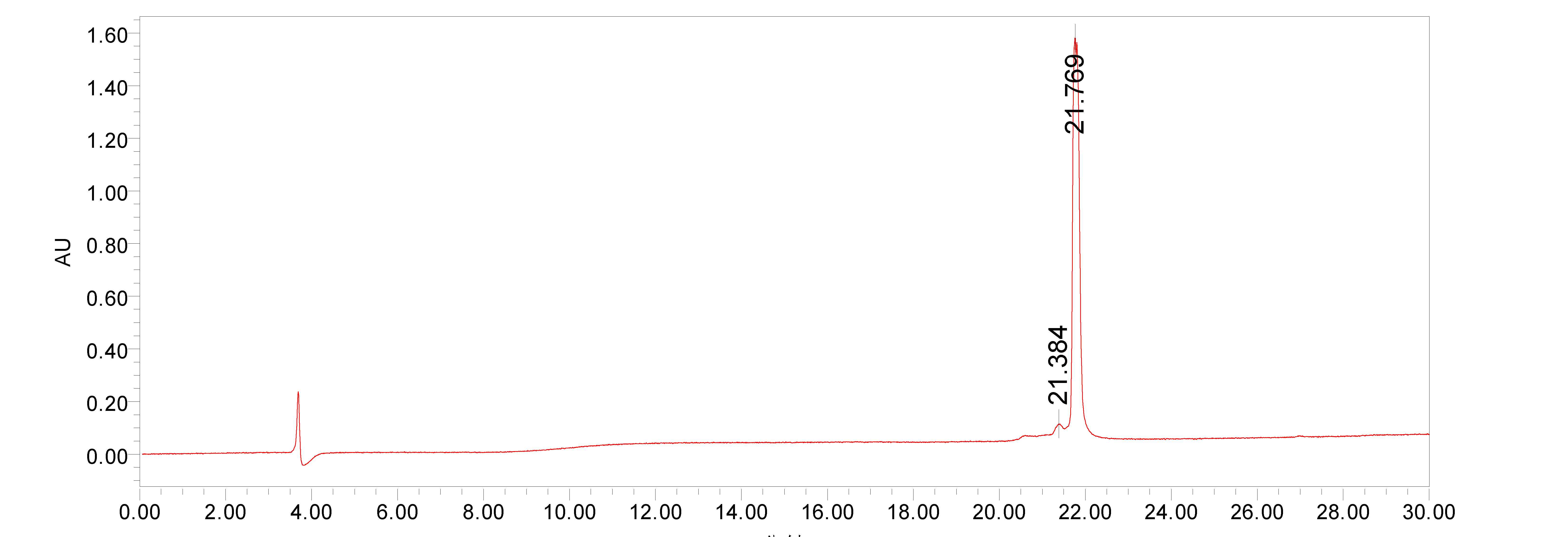


MS:


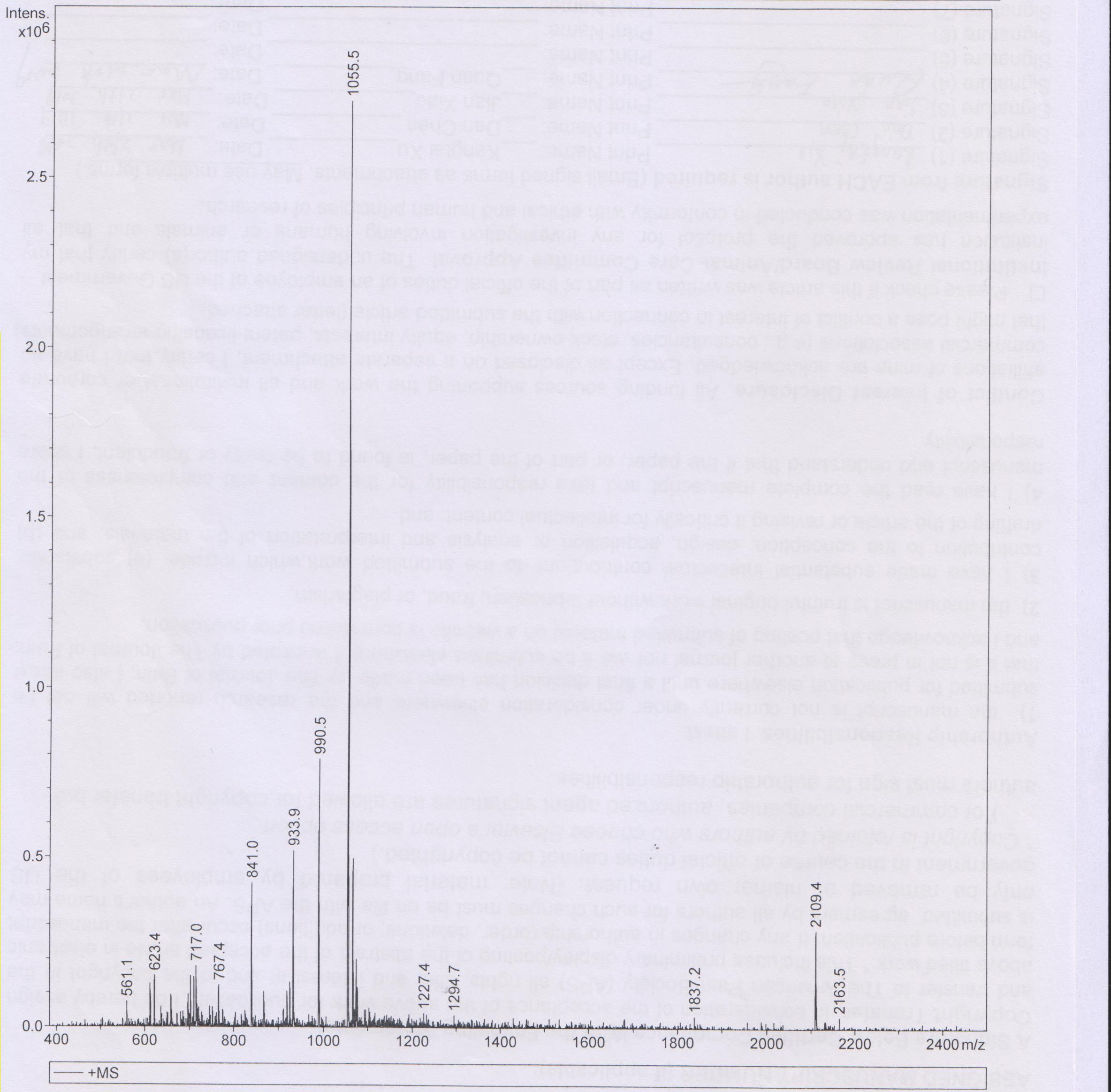

Supplement: Supplemental Material [file IDRD_A_1706669_SM2232.docx]
